# Effects of music medicine on anxiety among primigravida mothers of preterm neonates: The CalM MOM randomised controlled trial protocol

**DOI:** 10.1371/journal.pone.0360324

**Published:** 2026-11-03

**Authors:** Bishnupriya Mukherjee, Tara Rajendran, Anjali Maria Augustine, Akshay Patil, Subham Kumar Mohanta, Nutan Kamath

**Affiliations:** 1 Department of Pediatrics, Kasturba Medical College Mangalore, Manipal Academy of Higher Education, Manipal, India; 2 Department of Music, Faculty of Fine Arts, Annamalai University, Chidambaram, Tamil Nadu, India; 3 Department of Psychology, York University, Toronto, Canada; 4 University Hospitals of Derby and Burton NHS Foundation Trust, Derby, United Kingdom; 5 Global Biometrics and Data Sciences, Bristol Myers Squibb, Hyderabad, Telangana, India; University Hospital Cologne: Uniklinik Koln, GERMANY

## Abstract

**Background:**

Preterm birth and Neonatal Intensive Care Unit (NICU) admission are significant stressors that heighten maternal anxiety. Although RCTs have reported that music-based interventions used alongside usual care (UC) are effective and low-cost in reducing maternal anxiety, evidence from India remains limited.

**Objective:**

Primary objective is to assess the impact of listening to recorded classical Indian Instrumental *Saraswati Veena* music (*Ragas Kalyani* and *Hindolam*) (30-minutes/day over 7-days) alongside UC on maternal anxiety (*STAI-state anxiety)* of primigravida women with preterm neonates in the NICU. Secondary objectives measure changes in anxiety (Perinatal Anxiety Screening Scale (PASS)), maternal-infant bonding, breast milk volume, and neonatal body weight.

**Method:**

This three-arm parallel-group, superiority RCT among primigravida mothers with anxiety (screened using PASS) of preterm neonates (30–36 weeks of gestation, admitted to NICU) delivered vaginally at Government Lady Goschen Hospital, Mangalore. The intervention arm will receive Music Medicine (MM), the comparator arm Nature Sounds (NS), and the control arm UC. Participant blinding is not feasible (auditory intervention), and they serve as their own outcome-assessors (self-reported questionnaires); investigators and the independent statistician will remain blinded. Personnel interacting with participants are blinded to study objectives; enrolment/allocation staff blinded to the randomization sequence, and assessment staff blinded to group identity.

Randomization will employ a STATA-generated, permuted stratified block design stratified by baseline anxiety severity and gestational age (Allocation concealment: Sequentially Numbered, Opaque, Sealed Envelopes; Allocation Ratio: 1:1:1).

Sample size (66/arm; total N = 198) was determined through a simulation-based power analysis (5,000 iterations, base-R), assuming an MCID of 4 (MM) and 2 (NS) on the STAI, residual SD of ~8, powered at 80% (α = 0.05). Blinded sample-size re-estimation will be conducted at 50% enrollment completion. Primary powered analysis will employ ANCOVA (baseline-adjusted), and Secondary (exploratory) analyses will utilize a linear mixed-effects model for longitudinal continuous data with time × group interaction.

**Trial registration:**

Clinical Trial Registry of India; CTRI/2025/06/088896; URL: https://ctri.nic.in/Clinicaltrials/pmaindet2.php?EncHid=MTM0Mzg0&Enc=&userName=

**Protocol version:** Version 03, July 07, 2026

## Introduction

While strengthening maternal health is a global priority reflected in the United Nations’s Sustainable Development Goals (SDGs) and the World Health Organisation’s (WHO) Global Strategy for Women’s, Children’s and Adolescents’ Health, perinatal mental health remains an under-researched area despite its impact on clinical outcomes when left untreated [1–7]. Maternal anxiety is a concern around the perinatal period, with an estimated 10% to 40% of postpartum women experiencing anxiety disorders [3,5,8,9]. The highest rates of anxiety are reported during the initial weeks following childbirth and are higher in low- and middle-income countries (LMICs) [5,10]. While there are no distinct diagnostic criteria for postpartum anxiety currently, some researchers describe it as symptoms of generalized anxiety disorder occurring within four weeks after childbirth [8,11–14]. Maternal anxiety is a recognised risk factor for depression and suicide, and in addition, it can also have a critical, long-term negative impact on the mother and child [5,8,10,15]. It is more prevalent among mothers of preterm and low birth weight babies who have been admitted to the Neonatal Intensive Care Unit (NICU) [16]. Preterm birth has been linked to poor psychological functioning in mothers and is associated with negative parental interactive behaviors with their infants [9,16–19]. Lack of maternal postpartum care can lead to impaired initial mother-infant interactions, causing developmental delays and issues with social interaction as the child grows older [20]. This highlights the need for adjunct interventions that can be initiated early alongside usual care to address postpartum anxiety in resource-limited Indian healthcare settings.

The WHO Health Evidence Network Synthesis Report 2019 reported that music-based interventions (MBIs) lower maternal blood pressure and improve mother-infant bonding [21]. Musical engagement is reported to influence oxytocin levels, which mediates social bonding and induces a state of calm, thereby reducing stress [22–27]. In addition, relaxing music lowers the levels of hypothalamic-pituitary-adrenal axis markers of stress [28]. A 2021 meta-analysis reported that MBIs can significantly reduce postpartum anxiety [29]. A randomised controlled trial (RCT) conducted in 2021 among 126 women reported that those who received Turkish classical music had lower postpartum anxiety scores (p < .001) [30]. Music therapy and music medicine are two distinct forms of MBIs that must not be used interchangeably [31–33]. While music therapy, as defined by the American Music Therapy Association, is an evidence-based use of music interventions to accomplish individualized goals within a therapeutic relationship, Music Medicine (MM) refers to listening to pre-recorded music offered by the healthcare provider [34,35]. India currently lacks national medical regulatory body-accredited music therapy training programs, leading to the absence of National Medical Commission-accredited music therapists in the country [36–38]. Thus, MBI research in India is best classified as ‘Music Medicine’, given the lack of accredited music therapy training and regulation [33,39].

This CalM MOM (Classical Music for Mothers to Overcome Maternal-Anxiety) RCT evaluates the effect of music medicine on reducing anxiety in primigravida mothers of preterm neonates in resource-limited settings, thereby addressing a major research gap. The findings may offer insights contributing to SDG Targets 3.4 (mental health promotion) and 3.8 (universal health coverage) [40]. The protocol follows the Standard Protocol Items for Randomised Trials (SPIRIT) guidelines 2025 (S1 File) [41].

Primiparity, younger maternal age, preterm birth, NICU admission, reduced social support, barriers in access to postnatal mental health care, stigma around mental health issues, limited autonomy in care-seeking related decisions, and lower socio-economic status are some of the risk factors contributing to poor perinatal maternal mental health in LMICs [6,7,9,42]. In addition, resource-limited hospital settings have inadequate formal mental health care. Given these constraints, there is a need for low-cost, non-pharmacological adjunct interventions to be part of the early postpartum maternal care, and which can be continued at home after discharge. Evaluating music medicine through an RCT offers a rigorous approach to assessing a promising intervention to improve maternal anxiety. We will use nature sounds as an active auditory non-musical comparator to determine whether any observed effects are specific to music or are due to general auditory stimulation. We will also have a usual care group as control, representing the current standard of care, without any additional auditory intervention. This allows for the assessment of music’s added effect beyond routine care. To the best of our knowledge, this is going to be the first RCT from India evaluating the effects of instrumental classical South Indian *‘Saraswati Veena’* music on maternal anxiety among mothers of preterm neonates.

## Materials and methods

**Ethics**: The study protocol was reviewed and approved by the Institutional Ethics Committee of Kasturba Medical College and Hospital, Mangalore, Approval number: IEC-KMCMLR-04/2025/173; amendments were approved on 21 January 2026 (Version 02) and 15 July 2026 (Version 03). Written informed consent will be obtained from participants. Permission will be obtained from the medical superintendent of the hospital to recruit the patients and conduct the RCT.

### Trial objectives—PICO

**Population (P):** Primigravida mothers of NICU-admitted preterm infants (30–36 completed weeks of gestation born through normal vaginal delivery), with anxiety screened using the Perinatal Anxiety Screening Scale (PASS) and who understand English or Kannada.

**Intervention (I):** 30 minutes of Music Medicine using Classical South Indian instrumental music *(Raga Kalyani* and *Hindolam)* played on the *Saraswati Veena* with Usual Care.

**Comparator (C):** 1. 30 minutes of non-musical nature sounds (sounds of waves, river stream, and forest) with Usual Care

2. Usual Care only

**Outcome (O):** Change in maternal anxiety measured pre- and post-intervention using the State-Trait Anxiety Inventory (STAI)

**Time point of main interest:** Day 7 post-intervention (Fig 1)

**Fig 1:**
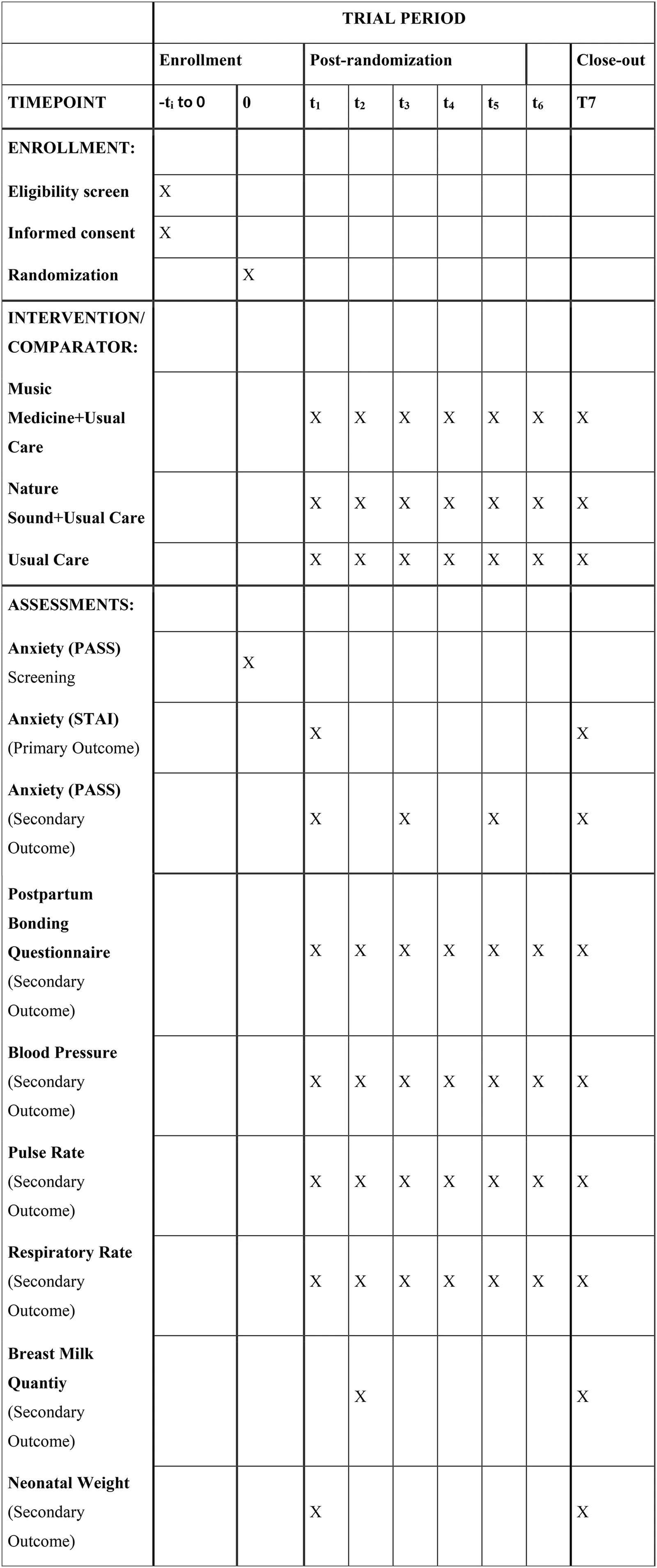
Participant timeline: Schedule of enrollment, intervention/comparators, and assessments.

Trial estimands are provided in S2 File.

### Design

3-arm, parallel-group, superiority RCT

### Study setting

Single-site, tertiary care teaching hospital—Government Lady Goschen Hospital, Kasturba Medical College, Mangalore, Karnataka, India. A designated private room with a quiet and supportive ambience, located within the NICU.

**Study period:** March 2025-December 2027

A) Participant recruitment: 15 September 2026
B) Data collection: 15 September 2026
C) Results are expected: December 2027

### Inclusion criteria

Primigravida mothers of NICU-admitted preterm infants (30–36 completed weeks of gestation born through normal vaginal delivery), with anxiety screened using the Perinatal Anxiety Screening Scale (PASS) and who understand English or Kannada.

### Exclusion criteria

Mothers with known psychiatric illness, severe or profound hearing loss, postpartum complications requiring intensive care, preterm twins or neonates with major congenital malformations or requiring invasive ventilatory support, prior experience with yoga or other relaxation techniques, or professional singing/musicianship will be excluded.

### Sampling method

Stratified random sampling

### Randomisation

**Method:** The study statistician will use permuted stratified block randomisation (block size 2,4, 6,8) stratified on baseline anxiety severity and gestational age (dichotomised).

**Random sequence generation:** A computerized random number generator in STATA will be used by the medical statisticians to ensure a reproducible and unbiased randomization process

**Allocation ratio:** 1:1:1

**Unit of randomization:** Individual mother.

The varying block sizes and sequence generation details will be documented in a separate, confidential file, inaccessible to those enrolling participants, to reduce the predictability of allocation.

### Allocation concealment

The random allocation sequence will be implemented using sequentially numbered, opaque, sealed envelopes (SNOSE) to ensure allocation concealment [43]. The envelopes will be prepared by the Principal Investigator (PI), who is not involved in participant recruitment, intervention delivery, or data analysis. Each envelope will contain a coded chit (e.g., Group A, B, or C), corresponding to one of the three arms. An independent hospital staff member, who is masked to study objectives and is not part of the research team, will randomly assign identities to Groups A, B, and C using a one-time draw from three sealed, identical envelopes. This process will be witnessed by two individuals and documented in a signed and dated form. The group identity key will be securely stored and not shared with the research team until the end of data collection.

To maintain allocation concealment:

- The independent hospital staff member who is responsible for screening and enrolling participants will not have access to the randomization sequence and allocation details.
- After obtaining written informed consent, the independent staff will hand over the sealed envelope to the participant.
- The participant will open the envelope only 15 minutes before the intervention session and sign it with the date.

This mechanism ensures that allocation concealment is maintained up to the point of assignment, thereby preventing selection bias and preserving the internal validity of the trial.

### Access to the random allocation sequence and assignment of roles

- **Access to the random allocation sequence:** The RA and the study statistician.
- **Who will enroll participants:** The independent hospital staff member will be responsible for identifying eligible participants and enrolling participants
- **Who will assign participants to their respective arms:** Post-enrolment, the independent hospital staff member will provide the sealed allocation envelope to the participant and will guide the participants to the designated room within the NICU. After the RA completes the initial assessments and leaves the room, the independent hospital staff member will select Music Medicine or Nature Sounds on the laptop. The participant will adjust the volume and chair–speaker distance to her comfort, and the staff will record these values in the monitoring sheet before exiting the room. After 30-minutes, the RA will enter and complete post-intervention assessments.
- **Ensuring blinding of enrollers and assigners:** The independent hospital staff will not have access to the random allocation sequence. RA will not have access to the identities of the groups. The randomization list will be stored in an encrypted file by the study statistician. The sealed envelopes will be securely stored in a locked cabinet in the Department of Pediatrics and accessed sequentially only after a participant is enrolled.

These measures ensure strict allocation concealment and prevent personnel directly interacting with participants from having any foreknowledge of the upcoming group assignments.

### Blinding

In this trial, the PI, co-PI, statisticians, RA, and faculty supervisor will be blinded to group identities. Due to the nature of the intervention (auditory experience), participants cannot be masked. Additionally, since the outcome measure is a participant-reported anxiety questionnaire, the participant also serves as the outcome assessor and therefore cannot be masked. The independent hospital staff member who directly interacts with the participant will be blinded to the study objectives; they will conduct the enrollment but will not have access to the random allocation sequence. The RA will be blinded to the study objectives and group identity.

The independent hospital staff member will prepare the blinded analysis dataset (removing treatment allocation) and will share it with the independent statistician. The independent statistician will be fully blinded and will perform the blinded sample size re-estimation (BSSR) analysis on blinded data only. He will fit the blinded analysis of covariance (ANCOVA) model and extract the residual variance (Mean Squared Error (MSE)), calculate the observed attrition rate, apply the pre-specified re-estimation formula, prepare the blinded BSSR report with the recommended sample size, and present the results to the Trial Steering Committee (TSC). The TSC will consist of independent members. The study statistician will remain blinded to the BSSR results after the TSC decision. He will update the statistical analysis plan only after TSC approval. Trial Management Group (TMG) will be blinded until the TSC decision, and it will coordinate logistics for the BSSR but will not access BSSR results before the TSC decision. TMG will implement operational changes only after TSC approval. It will review the BSSR reports from the independent statistician, make a final decision on sample size adjustments, and approve communication of any change to the trial team.

### Simulation-based sample size estimation

- **Primary powered analysis:** ANCOVA on STAI change from baseline to Day 7 (adjusting for baseline).
- **Secondary/exploratory:** A linear mixed-effects model for analysing longitudinal continuous data — with time × group interaction (no formal power estimation for secondary exploratory endpoints)

### Sample Size Justification – Simulation-Based Power Analysis (Evidence in Governance and Politics (EGAP) methodology)

We conducted a transparent simulation-based power analysis (5,000 iterations) for the primary ANCOVA contrast (Music vs Nature sounds vs Control, Minimal Clinically Important Difference (MCID) = 4 points for Music Medicine, MCID = 2 points for Nature Sounds, residual standard deviation (SD) ≈ 8, baseline adjustment).

**Complete R Code Used for Revised Power Calculation (Base R – EGAP style)**

*The following self-contained base-R script was used to generate the power estimates above:*

set.seed(123)

possible_n_per_arm <- seq(20, 100, by = 2)

powers < - rep(NA, length(possible_n_per_arm))

alpha < - 0.05; sims < - 5000

for (j in 1:length(possible_n_per_arm)) {

 n_per_arm < - possible_n_per_arm[j]; N < - n_per_arm * 3

 significant < - rep(NA, sims)

 for (i in 1:sims) {

  baseline < - rnorm(N, 45, 10)

  Y0 < - baseline + rnorm(N, 0, 8) # Control

  Y_music < - Y0 - 4; Y_nature < - Y0 - 2 # Effects (no direct reference: Nature Sounds comparator would have roughly half the effect of the Music Medicine intervention, will be revised during SSR)

  Z < - sample(c(“Control”,”Music”,”Nature”), N, TRUE, c(1/3,1/3,1/3))

  Y < - ifelse(Z==“Music”, Y_music, ifelse(Z==”Nature”, Y_nature, Y0))

  fit < - lm(Y ~ Z + baseline)

  p < - summary(fit)$coefficients[“ZMusic”, “Pr(>|t|)”]

  significant[i] <- (p <= alpha)

 }

 powers[j] <- mean(significant)

 }

data.frame(n_per_arm = possible_n_per_arm, power = round(powers, 3))

Therefore, a sample size of 66 participants per arm (total sample size = 198) (Fig 2) will be considered to achieve adequate power for the primary endpoint [44–50].

**Fig 2:**
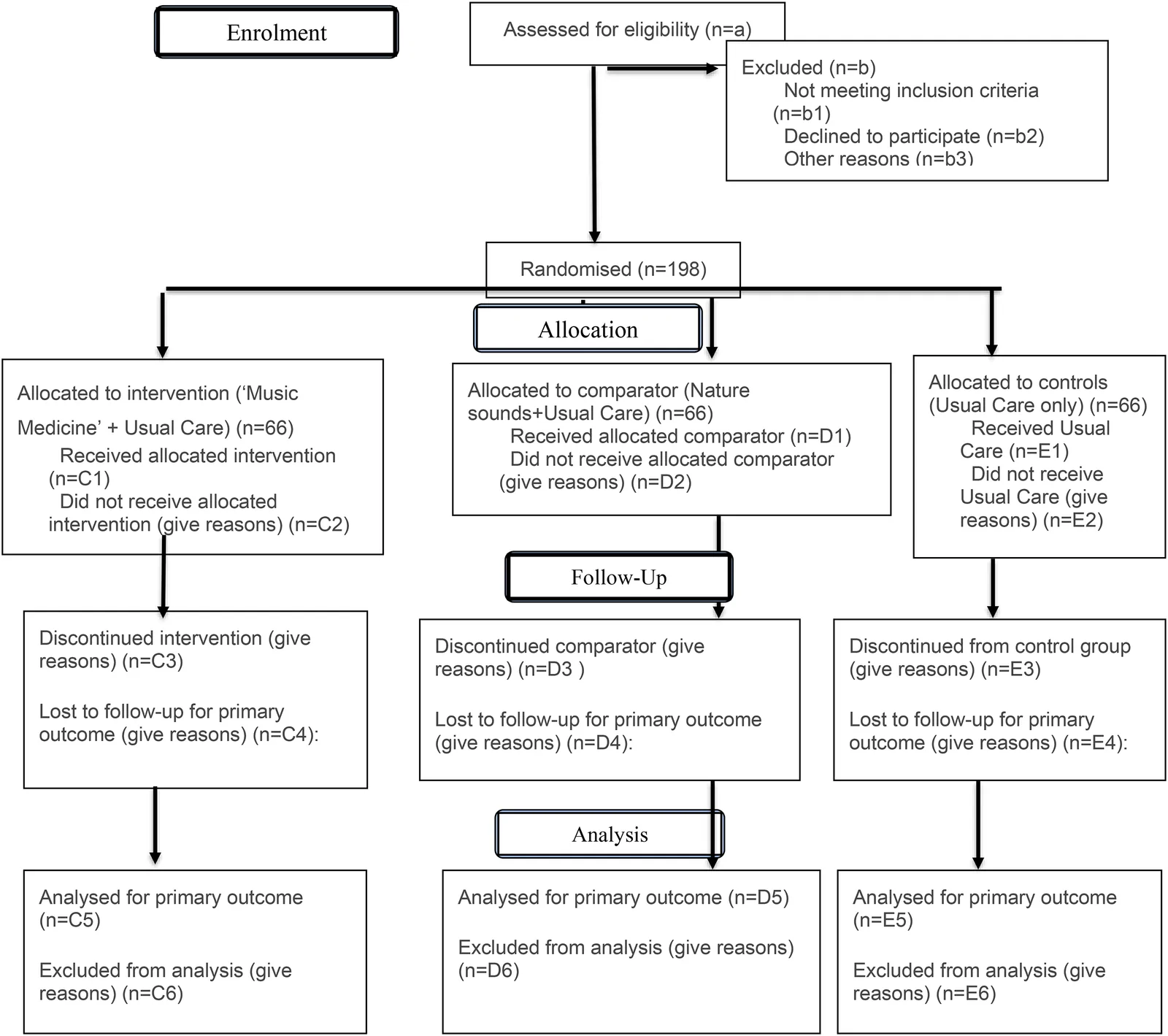
CONSORT 2025 flow diagram. Flow diagram of the progress through the phases of a randomised trial of **three arms** (that is, enrolment, intervention allocation, follow-up, and data analysis).

### Blinded Sample Size Re-estimation (BSSR) Plan & Recruitment Forecast

#### Internal assessment with BSSR.

**Rationale:** The internal assessment serves a dual purpose: (1) to validate key assumptions (recruitment, attrition, variability), and (2) to enable BSSR to ensure the trial remains adequately powered for the primary endpoint (change in STAI state anxiety at Day 7, MCID = 4 points). This approach follows best practice for adaptive designs with blinded re-estimation [49].

#### Objectives of the Internal assessment and BSSR.

- Validate recruitment rate and reasons for non-participation.
- Estimate the residual variance (SD) of the primary outcome (STAI change/post score adjusted for baseline).
- Estimate observed attrition/non-compliance rate at Day 7.
- Re-estimate the required total sample size to achieve 80% power for MCID = 4 points using blinded data.
- Adjust the final recruitment target (if necessary) while maintaining blinding.

#### Timing of the Internal Assessment/Interim BSSR Analysis.

**Timing:** After 50% of the target dataset has completed the Day 7 assessment.

For the target of 198 participants, this corresponds to approximately 99 participants completing their Day 7 assessment.

**Rationale for 50% timing:** This provides substantially more data for accurate estimation of residual variance, observed effect size, and attrition rate compared to an earlier look, leading to more reliable sample size re-estimation.

**Communication:** Results of the BSSR will be communicated to the TSC in a blinded manner. No unblinded efficacy comparisons will be performed.

### Data to be Used (Blinded)

The independent statistician will use only blinded data from participants who have completed Day 7:

- Baseline STAI state anxiety score
- Day 7 STAI state anxiety score
- Covariates pre-specified in the statistical analysis plan
- Treatment allocation will not be included in the model to maintain blinding.

### Methodology–BSSR

**Step 1: Model Fitting (Blinded ANCOVA)**

Fit an ANCOVA model on all available blinded data:


$$\begin{array}{c} \text{lm}(\text{Day7}\_\text{STAI }\sim \text{ Baseline}\_\text{STAI }+\text{ Covariates}) \end{array}$$

Obtain the MSE from this model. This is the blinded estimate of residual variance (σ^2^).

**Step 2: Estimate Attrition/Non-Compliance Rate**

Calculate the proportion of randomized participants who did not complete the Day 7 assessment (excluding those who have not yet reached Day 7). This gives the observed attrition rate (π).

**Step 3: Blinded Sample Size Re-estimation Formula**

We will use the methodology of Friede & Kieser (2011) for BSSR in ANCOVA designs [49]. The re-estimated total sample size (N*) required to achieve 80% power for MCID = 4 points is calculated as:


$$\begin{array}{c} {\text{N}}^{*}=\text{2}\times {\left((\text{Z}\_\{\text{1}-\alpha /\text{2}\}+\text{ Z}\_\{\text{1}-\beta \}\right)}^{\text{2}}\times \sigma^{\text{2}})/\delta^{\text{2}})\times (\text{1}/(\text{1}-{\text{R}}^{\text{2}})) \end{array}$$

Where:

- σ^2^ = Blinded estimate of residual variance from ANCOVA (MSE)
- δ = MCID = 4 points (Music Medicine) and 2 points (Nature Sounds)
- R^2^ = Proportion of variance explained by baseline STAI (typically 0.4–0.6)
- Z values for 80% power and two-sided α = 0.05

**Step 4: Adjust for Observed Attrition**

The final recruitment target (N_recruit) is calculated as:


$$\begin{array}{c} \text{N}\_\text{recruit }={\text{N}}^{*}/(\text{1}-\pi \_\text{observed}) \end{array}$$

Where π_observed is the attrition rate observed in the internal assessment.

**Step 5: Decision Rules (Updated for Target 198)**

The TSC will apply the following rules based on the re-estimated N_recruit (Table 1 and 2).

**Table 1:** Decision rule for blinded sample size re-estimation.

Re-estimated N_recruit	Action
≤ 180	Continue to revised target of 198 (no further increase needed)
181–220	Increase target to re-estimated N_recruit (TSC approval required)
> 240 or recruitment impossible	TSC to review feasibility; may cap at 220 or accept lower power with justification

**Table 2:** Recruitment Scenarios for Target = 198.

Scenario	Recruited/ month	Time to 198 participants	Feasibility & Mitigation
Optimistic	25–28	7–8 months	Requires dedicated RA + strong engagement
Realistic (Recommended)	18–22	9–11 months	Achievable with current resources + BSSR buffer
Conservative	12–15	13–16.5 months	Requires mitigation (extra support or timeline extension)

### Impact on statistical inference

Because the BSSR uses only blinded data and does not involve any comparison between treatment arms, it does not inflate the Type I error rate. The primary analysis will proceed as pre-specified in the SAP using ANCOVA on the final dataset. This is consistent with regulatory guidance on blinded adaptive designs.

Planned strategies to promote adequate enrolment are provided in S3 File.

### Intervention details—Music Medicine with usual care

This section is developed as per the Reporting Guidelines for Music-based Interventions checklist 2025 by Robb et al. and Pointers Specific to Classical Indian Music 2025 by Rajendran et al. (S4 File) [33,51].

### Intervention theory

A 2015 Indian study conducted among 29 mothers of premature infants, who received 30 minutes of Music Medicine using North Indian classical flute renditions of *Ragas* *Malkauns* and *Yaman* for four days, reported a significant increase in breast milk expression (p = 0.033) [52]. We will be using the analogous South Indian classical *Ragas ‘Hindolam’* and *‘Kalyani’* on a South Indian stringed instrument.

### Music selection

*Ragas* analogous to the 2015 study in South Indian classical music were chosen by a professional musician (co-PI) [52].

### Music

The musical recording to be used as the intervention of the RCT is validated by three music professors and professional musicians, and the recording is copyrighted with the Government of India (Certificate No.:SR-2025023565, Diary Number: SR-16851/2025-CO).

This recording comprises 29 minutes and 40 seconds of instrumental non-lyrical South Indian classical music in *Ragas Kalyani* and *Hindolam*, performed on the *Saraswati Veena*, a fretted acoustic instrument, by Tara Rajendran (Table 3). *Raga Hindolam* is a pentatonic scale, with five *swaras* (notes) in both ascending and descending scales, and *Raga Kalyani* contains all seven notes. The improvisational nature of classical Indian music makes the rendition of the *Ragas* unique to the artist. The structure of the recording is provided in Table 3. The music is accompanied by a digital *Tanpura*/drone from *Radel*. No percussive accompaniment is used, and the *Veena* is tuned to the standard tonic reference (*Sa*) at approximately D# pitch (South Indian classical system). Music is recorded using Matched Pair Small-diaphragm M5 Compact 1/2“Condenser Røde microphones and Presonus Studio 26 C, 2x4, 192 kHz, USB-C® Compatible Audio Interface; and audio editing is done using Garageband. All participants in the Music Medicine group will listen to the same music, with no individual tailoring or customization.

**Table 3:** Structure of Music Medicine intervention.

No.	Time stamp	Content
1	0:00–5:30	*Alaapana* (melodic improvisation) in *Raga Kalyani* characterised by free-flowing, non-metrical elaboration of the *Raga’*s melodic identity.
2	5:34–15:50	*Taana* (a semi-rhythmic melodic development) in *Raga Kalyani* featuring melodic patterns with varying tempos
3	15:57–20:44	*Alaapana* in *Raga Hindolam*
4	20:45–29:40	*Taana* in *Raga Hindolam*
	Total: 29 minutes 40 seconds	

### Music delivery method

Recorded music will be delivered through a small speaker connected to a laptop. Participants will adjust and control the volume to a level that is comfortable for them. The device’s built-in volume controls will be used to regulate the decibel level.

### Intervention materials

**Music materials:** The audio recordings used in the intervention.

**Non-music materials:** Speaker connected to a laptop. A comfortable chair for the participant to sit.

### Intervention strategy

Music listening

### Interventionist

This is a Music Medicine intervention; therefore, the music used will be pre-recorded, and no musician will be physically present in the designated private room. This music is performed and recorded by the co-PI, a professional *Sarawati Veena* musician with over a decade of performing experience. She has received 15 years of traditional training and also holds a Bachelor of Arts and Master of Fine Arts in *Sarawati Veena*, as well as a Ph.D. in music.

An independent hospital staff member masked to the objectives of the RCT, will turn on the music recording. After turning on the music on the speaker connected to the laptop, the staff member will exit the room, leaving the participant to listen to the music alone. The designated staff member will hold at least a bachelor’s degree.

### Unit of delivery

Intervention will be delivered to the individual participant

### Setting

A designated private room with a quiet ambience within the NICU of Government Lady Goschen Hospital, Kasturba Medical College, Mangalore, Karnataka, India.

**Intervention delivery schedule:**

Total number of sessions: 7

Session duration: 29 minutes and 40 seconds

Session frequency: Once a day

Time: Between 8 am and 5 pm

### Intervention fidelity

Single interventionist. A checklist will be provided to ensure fidelity.

### Comparator details—Nature sounds with Usual Care

The comparator will be Nature Sounds (Table 4) with Usual Care. Structure, components, duration, and source are listed in Table 4.

**Table 4:** Non-musical Nature Sounds.

No.	Title	Time stamp	Source	Author details
1	***Sound of Waves*** https://pixabay.com/sound-effects/sounds-of-waves-313367/	0.00–13.55 Minutes	Pixabay (Free for use under the Pixabay Content License)	MindMist
2	***Running stream/water sounds*** https://pixabay.com/sound-effects/running-stream-water-sound-239612/	13.56–23.11 Minutes	Pixabay (Free for use under the Pixabay Content License)	AllyInNature
3	***Forest ambience morning,spring, localization Poland 6*** https://pixabay.com/sound-effects/forest-ambience-morningspring-localization-poland-6-296922/	23.11-24.23 Minutes	Pixabay (Free for use under the Pixabay Content License)	AudioPapkin
4	***River Stream (Moderate Flow 2)*** https://pixabay.com/sound-effects/river-stream-moderate-flow-2-24370/	24.24—26.31 Minutes	Pixabay (Free for use under the Pixabay Content License)	CaganCelik (Freesound)
5	***Forest ambience*** https://pixabay.com/sound-effects/forest-ambience-296528/	26.32—29-39 Minutes	Pixabay (Free for use under the Pixabay Content License)	AudioPapkin
		Total: 29 minutes 39 seconds		

### Criteria for discontinuation of intervention/comparator

- **Participant request:** If the mother expresses discomfort, distress, or unwillingness to continue Music Medicine or Nature Sounds at any point, the session will be stopped immediately.
- **Clinical deterioration of mother or neonate:** If either the mother or the neonate experiences any clinical deterioration requiring urgent medical intervention, the session will be discontinued.
- **No forced continuation:** Participants will be clearly informed that the participation can be withdrawn at any time and that will never affect their or their infant’s medical care.

### Criteria for modification of intervention/comparator

No modification is planned.

### Usual Care

All arms (Music Medicine arm, Nature sounds arm, and the Usual Care arm) will receive usual care, and that is the standard postnatal care routinely provided by the hospital, which includes but is not limited to:

- Routine clinical monitoring of the mother and neonate by pediatric and nursing staff
- Antibiotics, if clinically indicated
- Kangaroo Mother Care (KMC) as per guidelines
- Breastfeeding support and guidance
- Access to counseling services if requested
- Standard postpartum education provided by the hospital team about essential newborn care

In the Usual Care arm, no additional interventions will be administered beyond the usual care.

### Tracking of usual care

Logs will be maintained of the usual care received by each participant, as well as any other significant medical or supportive interventions provided.

### Usual care in music medicine and nature sounds arms

Participants in the Music Medicine and Nature Sounds arms will continue to receive the same usual care as outlined above. Music Medicine and Nature Sounds are adjunctive and will not substitute for or interfere with any element of routine care.

### Strategies to improve adherence

- **Standard operating procedure:** Independent hospital staff member will follow a standardized procedure to set up the session, ensuring uniform delivery across all participants.
- **Supportive environment:** Sessions will be conducted in a designated room within the NICU, a quiet and comfortable space that’s completely private, minimizing distractions and enhancing engagement.
- **Rapport building and participant engagement:** To enhance comfort and trust, the personnel directly interacting with the participants will engage in brief rapport-building conversations with each mother on the day of childbirth, before pre-intervention assessments. This step is expected to foster better participant engagement and improve session attendance and adherence throughout the trial.

### Monitoring adherence to intervention/comparator protocols

To ensure adherence and fidelity to the intervention protocol, the independent hospital staff member will use checklists with each participant.

#### Permitted concomitant care.

All routine medical and nursing interventions that are part of Usual Care

#### Prohibited concomitant care:.

- Additional music or sound-based interventions during the RCT.
- Additional relaxation practices during the RCT

### Documentation

All concomitant care activities will be documented in the monitoring chart.

### Harms

Definition and assessment of harms, along with a checklist, are provided under S5 Files.

### Strategies to minimise contamination between study arms

As the study will be conducted in a resource-limited setting, dedicated facilities to accommodate participants from different study arms in separate rooms will not be available, and mothers will continue to reside in the same postnatal wards. However, to minimise contamination between the three arms, the following strategies will be implemented:

- **Private study setting:** All 30-minute intervention sessions (Music Medicine and Nature Sounds) will be conducted in a designated quiet private room separate from the shared wards. Participants in the usual care arm will receive no audio intervention.
- **Individual staff supervision:** An independent hospital staff member, masked to the study objectives, will escort each participant to the intervention room, activate only the arm-specific audio file on the laptop, and then leave the room. The RA will conduct pre- and post-session assessments but will not be present during intervention delivery.
- **Sequential scheduling:** As participants from different study arms reside in the same postnatal wards, intervention sessions will be individually scheduled and conducted sequentially to minimise contact between participants undergoing different interventions. Real-time coordination will be maintained through recruitment and intervention logs.
- **Participant instructions:** During the informed consent process, participants will be explicitly instructed not to discuss the content or details of their sessions with other mothers in the ward.
- **Fidelity documentation:** Monitoring charts (S6 File) will record the exact session time, decibel level, and speaker-to-participant distance for every intervention session to ensure protocol adherence.

### Tools

- Perinatal Anxiety Screening Scale (PASS) [53]. The tool is translated into the *Kannada* language and validated with permission from the owners
- Post-enrollment proforma (neonatal clinical details and the maternal profile, including socioeconomic status assessed using the Modified *Kuppuswamy* Scale). The proforma has been validated.
- State-Trait Anxiety Inventory (STAI) *Kannada*.
- Postpartum Bonding Questionnaire (PBQ) [54]. The tool is translated into the *Kannada* language and validated.
- Post-intervention questionnaire (on day 8 after the intervention). The tool is validated.

### Outcomes

#### Primary outcome.

**Mean change in maternal anxiety scores as measured (pre- and post-intervention) by the State-Trait Anxiety Inventory (STAI) from baseline (Day 1) to Day 7.**

**Rationale**: Anxiety in mothers of preterm neonates in the NICU is common and can negatively impact maternal well-being and infant care. STAI is a well-validated questionnaire that measures anxiety levels, distinguishing between *state anxiety* (anxiety about an event/situation, which in this case is the NICU experience) and *trait anxiety* (general predisposition).

#### Secondary outcomes.

- **Mean change in maternal anxiety scores as assessed by the Perinatal Anxiety Screening Scale (PASS) pre- and post-intervention from baseline (Day 1) to Day 7.**

**Rationale:** PASS for assessing anxiety specific to the perinatal period. It includes factors such as the neonate’s health, NICU admission, and breastfeeding concerns, all of which are closely linked to the study and are therefore relevant to outcome generation.

- **Mean change in the Postpartum Bonding Questionnaire (PBQ) scores as measured (pre-intervention) from baseline (Day 1) to Day 7 (post-intervention).**

**Rationale:** Multiple RCTs and meta-analyses support a positive correlation between music exposure and increased oxytocin levels.

- **Mean change in the volume of breast milk expressed on Day 2 and Day 7.**

**Rationale:** Maternal anxiety and NICU separation can impair milk production, while music has been shown to increase oxytocin (facilitating milk letdown), reduce cortisol (stress hormone), and, as demonstrated by RCTs, enhance both breast milk volume and breastfeeding frequency.

- **Mean change in maternal vital signs (blood pressure, pulse rate, and respiratory rate) measured pre- and post-intervention on Day 1 and Day 7.**

**Rationale**: Physiological parameters such as blood pressure, respiratory rate, and pulse rate serve as indicators of autonomic nervous system response, and various RCTs and meta-analyses report that music-based interventions modulate these measures.

- **Mean change in neonatal weight in grams on Day 1 and Day 7.**

**Rationale**: Neonatal weight is included as a secondary outcome to explore the indirect impact of the maternal music intervention on infant health, specifically through its potential effects on maternal lactation and caregiving behaviors. Preterm neonates typically lose weight during the first 10 days, and therefore, we will document the percentage of weight loss in all three groups.

**Core outcome set:** No core outcome set is available for maternal anxiety in mothers of preterm neonates; outcomes were selected based on clinical relevance and literature.

### Data collection

The mode of data collection in this RCT will be paper-based. The screening procedure will be done by an independent hospital staff member following the eligibility checklist and preliminary PASS screening test. They will be trained for weeks before trial commencement under the supervision of the PI and will follow a standardized checklist to ensure consistency.

RA will provide STAI pre-intervention (Days 1 and 7), PASS (Days 1,3,5,7), PBQ (all 7 days) to the participants to answer and measure physiological parameters (blood pressure, respiratory rate, pulse rate) (all 7 days) and leave the room. Following the participant’s adjustment of the decibel and seat position, the independent staff member will document the decibel level and the distance between the speaker and the participant. The RA will enter the room after 30 minutes and will proceed to measure the post-intervention physiological parameters (all 7 days), and then provide the STAI (days 1 and 7), PASS questionnaires (Days 1,3,5,7), and PBQ (all 7 days) to the participants. RA will collect all the questionnaires.

Breast milk volume will be measured using a *Medela* breast pump and a graded container on day 2 and day 7. Monitoring charts for intervention and control arms are provided in S6 File.

### Data management

All data will be anonymized. Data will be entered independently by both the RA and the independent staff member. Electronic data will be stored in password-protected files. Physical documents will be stored in locked cabinets in the pediatrics department office. All data will be retained for a minimum of 5 years post-publication, in accordance with institutional and regulatory guidelines.

### Statistical analysis plan

Baseline characteristics will be summarized using appropriate summary statistics. Continuous variables will be presented as means and standard deviations, while categorical variables will be described using frequencies and percentages.

The assumptions of normality and homogeneity of variances will be checked before conducting parametric tests. The Shapiro-Wilk test will be used to assess normality, and Levene’s test will be applied to evaluate the homogeneity of variances. If violated, a non-parametric alternative like the Van Elteren test (stratified Wilcoxon rank-sum) will be applied, stratifying by baseline anxiety levels and gestational age. The primary and secondary analysis plan is provided in detail below. For repeated measures (e.g., vital signs, breast milk volume over time), mixed-effects models will be employed, with fixed effects for group and time, and random effects for participants. Post-hoc tests will use Bonferroni correction. All analyses will be conducted using R, and p < 0.05 is considered significant.

### Analysis population

Baseline characteristics will be presented for all randomised participants with at least one post-baseline STAI assessment.

#### Primary endpoint analysis.

The primary outcome is the change in STAI state anxiety score from baseline (Day 1 pre-intervention) to Day 7 post-intervention.

The primary analysis will be an ANCOVA comparing the mean change in STAI scores between the three groups, adjusting for baseline STAI score. This analysis corresponds to the pre-specified powered primary hypothesis.

**Assumption checking and alternative analysis:** Prior to analysis, normality of residuals and homogeneity of variances will be assessed. If these assumptions are violated, the Van Elteren test (stratified Wilcoxon rank-sum test) will be used as the primary non-parametric analysis, stratified by baseline anxiety level and gestational age (dichotomised at the median or clinically relevant cut-off).

If the overall test is statistically significant, post-hoc pairwise comparisons will be performed using Bonferroni correction.

#### Secondary endpoint analyses.

All secondary endpoint analyses are considered exploratory. No adjustment for multiplicity will be applied.

- **Perinatal Anxiety Screening Scale**: A mixed-effects model for repeated measures (MMRM) or repeated measures ANCOVA will be used to compare trajectories over time (Days 1, 3, 5, and 7), adjusting for baseline score where appropriate. Group × time interaction will be explored.
- **Postpartum Bonding Questionnaire**: ANCOVA or MMRM will be used to compare total and subscale scores, with adjustment for baseline score if relevant.
- **Breast Milk Volume (Day 2 and Day 7)**: ANCOVA on Day 7 values adjusting for Day 2 measurement, or MMRM if appropriate.
- **Neonatal Body Weight (Day 1 and Day 7)**: ANCOVA on Day 7 weight, adjusting for baseline weight and relevant covariates (e.g., gestational age at birth).
- **Vital Parameters (Blood Pressure, Pulse Rate, Respiratory Rate)**: Within-group pre-post changes will be assessed using paired t-tests. Between-group comparisons at individual time points will use ANOVA or ANCOVA. Time-by-group interaction effects will be explored using MMRM. These analyses are exploratory.

### Missing data and sensitivity analysis

The primary method for handling missing data will be Multiple Imputation (MI), under the assumption that the data is Missing at Random (MAR). To assess the robustness of findings, sensitivity analyses will be conducted using the Last Observation Carried Forward (LOCF) and complete-case analysis. All analyses will adhere to the intention-to-treat (ITT) principle, including all randomized participants as allocated.

All statistical analyses will be performed using R software. A two-sided p-value of less than 0.05 will be considered statistically significant.

## Discussion

The development of the CalM MOM RCT protocol has been informed by existing literature on postnatal maternal anxiety and prior trials that reported positive results of Music Medicine on maternal anxiety. The intervention parameters (the selection of *Ragas* *Kalyani* and *Hindolam* and the 30-minute duration) were refined based on published evidence. In addition, the musical recording used for the intervention was certified by three independent professors and professional musicians for authenticity and suitability. The detailed structure and content of musical recording are provided according to the Reporting Guidelines for Music-Based Intervention Guide 2025 and Pointers for classical Indian music-based interventions 2025. The feasibility of recruitment, data collection, and logistics was evaluated in consultation with clinical faculties at the study setting. The protocol has undergone methodological appraisal by the institutional ethics committee and a team of medical statisticians. All sections have been developed in accordance with SPIRIT guidelines to ensure transparency, reproducibility, and methodological robustness.

The incorporation of an internal assessment will allow early evaluation of feasibility, recruitment, attrition, and variability in the primary outcome, ensuring that key assumptions underlying the sample-size calculation are appropriate for this population and setting. These data-driven adjustments will strengthen the methodological rigor and improve the reliability and efficiency of the RCT.

Due to the nature of the intervention, masking participants from group allocation will not be feasible; however, using nature sounds as a comparator and masking both the RA and independent statistician from group identity will help minimise bias [55]. The outcome assessor is the participant themselves, as the outcome measurements are through self-reported measures (STAI, PASS, and PBQ), and this might also introduce bias, although these instruments are appropriate and are commonly used in mental health research; in future trials, we will consider incorporating biomarker-based assessments to improve objectivity [55]. This will be a single-site study, allowing close monitoring and methodological consistency within available resources. Although the sample size and seven-day intervention period will be modest, they will provide an important foundation for generating preliminary evidence and refining procedures for subsequent multi-centre research of longer trial duration.

S7-S9 Files contains additional details and explanations as per SPIRIT guidelines (Administrative information (Roles and responsibilities and Individual trial participant data sharing statement); Monitoring (Data monitoring committee, Trial monitoring, Trial Management Group, Trial Steering Committee, Data Management Team, Endpoint Adjudication Committee); Ethics (Protocol amendments, written informed consent process), Confidentiality, Ancilliary and post-trial care, Patient and public involvement; Dissemination policy; Data sharing).

## Supporting information

S1 File Standard Protocol Items for Randomised Trials (SPIRIT) 2025 checklist of items to address in a randomized trial protocol. (DOCX)

S2 File Estimand Framework for the Primary Objective. (DOCX)

S3 File Planned strategies to promote adequate enrolment. (DOCX)

S4 File Reporting Guidelines for Music-based Interventions Checklist 2025 and Pointers specific to classical Indian music 2025. (DOCX)

S5 File Harms definition and checklist. (DOCX)

S6 File Monitoring charts-intervention arm and control arm. (DOCX)

S7 File Roles and Responsibilities of investigators. (DOCX)

S8 File Study Monitoring Groups. (DOCX)

S9 File Additional details. (DOCX)

## References

[1] Britton JR. Pre-discharge anxiety among mothers of well newborns: prevalence and correlates. Acta Paediatr. 2005;94(12):1771–6. doi: 10.1111/j.1651-2227.2005.tb01852.x 16421038

[2] Paul IM, Downs DS, Schaefer EW, Beiler JS, Weisman CS. Postpartum anxiety and maternal-infant health outcomes. Pediatrics. 2013;131(4):e1218-24. doi: 10.1542/peds.2012-2147 23460682

[3] Howard LM, Molyneaux E, Dennis C-L, Rochat T, Stein A, Milgrom J. Non-psychotic mental disorders in the perinatal period. The Lancet. 2014;384(9956):1775–88. doi: 10.1016/s0140-6736(14)61276-9 25455248

[4] Stein A, Pearson RM, Goodman SH, Rapa E, Rahman A, McCallum M, et al. Effects of perinatal mental disorders on the fetus and child. The Lancet. 2014;384(9956):1800–19. doi: 10.1016/s0140-6736(14)61277-0 25455250

[5] Dennis C-L, Falah-Hassani K, Shiri R. Prevalence of antenatal and postnatal anxiety: systematic review and meta-analysis. Br J Psychiatry. 2017;210(5):315–23. doi: 10.1192/bjp.bp.116.187179 28302701

[6] The Lancet. Perinatal depression: a neglected aspect of maternal health. Lancet. 2023;402(10403):667. doi: 10.1016/S0140-6736(23)01786-5 37633661

[7] Duffy M, Churchill R, Kak LP, Reap M, Galea JT, O’Donnell Burrows K, et al. Strengthening perinatal mental health is a requirement to reduce maternal and newborn mortality. Lancet Reg Health Am. 2024;39:100912. doi: 10.1016/j.lana.2024.100912 39507364 PMC11539155

[8] Pawluski JL, Lonstein JS, Fleming AS. The neurobiology of postpartum anxiety and depression. Trends in Neurosciences. 2017;40(2):106–20. doi: 10.1016/j.tins.2016.11.009 28129895

[9] Field T. Postnatal anxiety prevalence, predictors and effects on development: A narrative review. Infant Behav Dev. 2018;51:24–32. doi: 10.1016/j.infbeh.2018.02.005 29544195

[10] McNab SE, Dryer SL, Fitzgerald L, Gomez P, Bhatti AM, Kenyi E, et al. The silent burden: a landscape analysis of common perinatal mental disorders in low- and middle-income countries. BMC Pregnancy Childbirth. 2022;22(1):342. doi: 10.1186/s12884-022-04589-z 35443652 PMC9019797

[11] Jotzo M, Poets CF. Helping parents cope with the trauma of premature birth: an evaluation of a trauma-preventive psychological intervention. Pediatrics. 2005;115(4):915–9. doi: 10.1542/peds.2004-0370 15805364

[12] Goodman JH, Watson GR, Stubbs B. Anxiety disorders in postpartum women: A systematic review and meta-analysis. J Affect Disord. 2016;203:292–331. doi: 10.1016/j.jad.2016.05.033 27317922

[13] Zappas MP, Becker K, Walton-Moss B. Postpartum anxiety. J Nurse Practitioners. 2021;17(1):60–4. doi: 10.1016/j.nurpra.2020.08.017

[14] Zain E, Watanabe Y, Takabayashi S, Por L, Fujita S, Moriyama S, et al. Psychometric evaluation of the Japanese Edinburgh Postnatal Depression Scale for screening postpartum anxiety. Front Psychiatry. 2025;16:1659497. doi: 10.3389/fpsyt.2025.1659497 41069938 PMC12504224

[15] Zelkowitz P, Papageorgiou A. Maternal anxiety: an emerging prognostic factor in neonatology. Acta Paediatr. 2005;94(12):1704–5. doi: 10.1111/j.1651-2227.2005.tb01840.x 16421027

[16] Worrall S, Christiansen P, Khalil A, Silverio SA, Fallon V. Associations between prematurity, postpartum anxiety, neonatal intensive care unit admission, and stress. Front Psychiatry. 2024;15:1323773. doi: 10.3389/fpsyt.2024.1323773 38463430 PMC10921229

[17] Dunkel Schetter C, Tanner L. Anxiety, depression and stress in pregnancy: implications for mothers, children, research, and practice. Curr Opin Psychiatry. 2012;25(2):141–8. doi: 10.1097/YCO.0b013e3283503680 22262028 PMC4447112

[18] van der Zee-van den Berg AI, Boere-Boonekamp MM, Groothuis-Oudshoorn CGM, Reijneveld SA. Postpartum depression and anxiety: a community-based study on risk factors before, during and after pregnancy. J Affect Disord. 2021;286:158–65. doi: 10.1016/j.jad.2021.02.062 33725615

[19] Zheng W, Chotipanvithayakul R, Ingviya T, Guo F. Effects of home-based integrated sensory stimulation program to preterm infants on parents’ depression and anxiety: a randomized controlled trial. Glob Health Action. 2025;18(1):2491848. doi: 10.1080/16549716.2025.2491848 40314668 PMC12051557

[20] Beck CT. The effects of postpartum depression on child development: a meta-analysis. Arch Psychiatr Nurs. 1998;12(1):12–20. doi: 10.1016/s0883-9417(98)80004-6 9489170

[21] Fancourt D, Finn S. What is the evidence on the role of the arts in improving health and well-being? A scoping review. Regional Office for Europe: World Health Organization. 2019. 32091683

[22] Grape C, Sandgren M, Hansson L-O, Ericson M, Theorell T. Does singing promote well-being?: An empirical study of professional and amateur singers during a singing lesson. Integr Physiol Behav Sci. 2003;38(1):65–74. doi: 10.1007/BF02734261 12814197

[23] World Health Organization. Infant and young child feeding: model chapter for textbooks for medical students and allied health professionals. World Health Organization. 2025. 23905206

[24] Hansen NC, Keller PE. Oxytocin as an allostatic agent in the social bonding effects of music. Behav Brain Sci. 2021;44:e75. doi: 10.1017/S0140525X20001235 34588045

[25] Good A, Russo FA. Changes in mood, oxytocin, and cortisol following group and individual singing: A pilot study. Psychology of Music. 2021;50(4):1340–7. doi: 10.1177/03057356211042668

[26] Bowling DL. Biological principles for music and mental health. Transl Psychiatry. 2023;13(1):374. doi: 10.1038/s41398-023-02671-4 38049408 PMC10695969

[27] Busse PK, Neugebauer L, Kaschubowski G, Anheyer D, Ostermann T. Oxytocin as a physiological correlate of dyadic music therapy relationships - a randomized crossover pilot study. Front Behav Neurosci. 2025;18:1504229. doi: 10.3389/fnbeh.2024.1504229 39949817 PMC11821654

[28] Chanda ML, Levitin DJ. The neurochemistry of music. Trends Cogn Sci. 2013;17(4):179–93. doi: 10.1016/j.tics.2013.02.007 23541122

[29] Hakimi S, Hajizadeh K, Hasanzade R, Ranjbar M. A Systematic review and meta-analysis of the effects of music therapy on postpartum anxiety and pain levels. J Caring Sci. 2021;10(4):230–7. doi: 10.34172/jcs.2021.033 34849370 PMC8609126

[30] Toker E, Demirel G, Doganer A, Karakucuk S. Effects of Turkish classical music on postpartum pain and anxiety in cesarean deliveries: a randomized controlled trial. Altern Ther Health Med. 2021;27(S1):120–7. 33245708

[31] Robb SL, Hanson-Abromeit D, May L, Hernandez-Ruiz E, Allison M, Beloat A, et al. Reporting quality of music intervention research in healthcare: A systematic review. Complement Ther Med. 2018;38:24–41. doi: 10.1016/j.ctim.2018.02.008 29857877 PMC5988263

[32] Bradt J, Dileo C, Myers-Coffman K, Biondo J. Music interventions for improving psychological and physical outcomes in people with cancer. Cochrane Database Syst Rev. 2021;10(10):CD006911. doi: 10.1002/14651858.CD006911.pub4 34637527 PMC8510511

[33] Rajendran T, Patil A, Mohanta SK, Krishnadasa SN, Natarajan V. Classical Indian music for managing anxiety and pain among patients in a hospital setting: a systematic review and meta-analysis of randomized controlled trials. J Public Health (Oxf). 2025;47(3):527–39. doi: 10.1093/pubmed/fdaf048 40331588

[34] Bradt J, Dileo C, Shim M. Music interventions for preoperative anxiety. Cochrane Database Syst Rev. 2013;2013(6):CD006908. doi: 10.1002/14651858.CD006908.pub2 23740695 PMC9758540

[35] Bradt J, Dileo C, Magill L, Teague A. Music interventions for improving psychological and physical outcomes in cancer patients. Cochrane Database Syst Rev. 2016;(8):CD006911. doi: 10.1002/14651858.CD006911.pub3 27524661

[36] Rajendran T. Addressing the need for personalizing music therapy in integrative oncology. J Integr Med. 2022;20(4):281–3. doi: 10.1016/j.joim.2022.01.005 35078746

[37] Rajendran T. Music therapy in cancer care: unravelling the complexities in LMICs. Support Care Cancer. 2023;31(12):715. doi: 10.1007/s00520-023-08188-2 37991584

[38] Rajendran T. Neglected local soundscapes in music therapy: cultural equity in research, policy, and practice in integrative oncology. Support Care Cancer. 2025;33(12):1144. doi: 10.1007/s00520-025-10097-5 41313361

[39] Bradt J, Potvin N, Kesslick A, Shim M, Radl D, Schriver E, et al. The impact of music therapy versus music medicine on psychological outcomes and pain in cancer patients: a mixed methods study. Support Care Cancer. 2015;23(5):1261–71. doi: 10.1007/s00520-014-2478-7 25322972

[40] Minas H, Tsutsumi A, Izutsu T, Goetzke K, Thornicroft G. Comprehensive SDG goal and targets for non-communicable diseases and mental health. Int J Ment Health Syst. 2015;9:12. doi: 10.1186/s13033-015-0003-0 25774216 PMC4359436

[41] Chan AW, Boutron I, Hopewell S, Moher D, Schulz KF, Collins GS, et al. SPIRIT 2025 statement: updated guideline for protocols of randomised trials. The Lancet. 2025;405(10491):e19-27. doi: 10.1016/S0140-6736(25)00770-6 42290521

[42] Nisar A, Yin J, Waqas A, Bai X, Wang D, Rahman A, et al. Prevalence of perinatal depression and its determinants in Mainland China: A systematic review and meta-analysis. J Affect Disord. 2020;277:1022–37. doi: 10.1016/j.jad.2020.07.046 33065811

[43] Doig GS, Simpson F. Randomization and allocation concealment: a practical guide for researchers. J Crit Care. 2005;20(2):187–91; discussion 191-3. doi: 10.1016/j.jcrc.2005.04.005 16139163

[44] Faul F, Erdfelder E, Lang A-G, Buchner A. G*Power 3: a flexible statistical power analysis program for the social, behavioral, and biomedical sciences. Behav Res Methods. 2007;39(2):175–91. doi: 10.3758/bf03193146 17695343

[45] Julian LJ. Measures of anxiety: State-Trait Anxiety Inventory (STAI), Beck Anxiety Inventory (BAI), and Hospital Anxiety and Depression Scale-Anxiety (HADS-A). Arthritis Care Res (Hoboken). 2011;63 Suppl 11(0 11):S467-72. doi: 10.1002/acr.20561 22588767 PMC3879951

[46] Cohen J. Statistical power analysis for the behavioral sciences. Routledge. 2013. doi: 10.4324/9780203771587

[47] Norman GR, Sloan JA, Wyrwich KW. Interpretation of changes in health-related quality of life: the remarkable universality of half a standard deviation. Med Care. 2003;41(5):582–92. doi: 10.1097/01.MLR.0000062554.74615.4C 12719681

[48] Spielberger CD, Gorsuch RL, Lushene R, Vagg PR, Jacobs GA. Manual for the State-Trait Anxiety Inventory. Palo Alto, CA: Consulting Psychologists Press. 1983.

[49] Friede T, Kieser M. Blinded sample size recalculation for clinical trials with normal data and baseline adjusted analysis. Pharm Stat. 2011;10(1):8–13. doi: 10.1002/pst.398 19943322

[50] Singh VP, Rao V, V P, R C S, K KP. Comparison of the effectiveness of music and progressive muscle relaxation for anxiety in COPD--A randomized controlled pilot study. Chron Respir Dis. 2009;6(4):209–16. doi: 10.1177/1479972309346754 19858350

[51] Robb SL, Story KM, Harman E, Burns DS, Bradt J, Edwards E, et al. Reporting Guidelines for Music-based Interventions checklist: explanation and elaboration guide. Front Psychol. 2025;16:1552659. doi: 10.3389/fpsyg.2025.1552659 40567875 PMC12188939

[52] Ak J, Lakshmanagowda PB, G C M P, Goturu J. Impact of music therapy on breast milk secretion in mothers of premature newborns. J Clin Diagn Res. 2015;9(4):CC04-6. doi: 10.7860/JCDR/2015/11642.5776 26023551 PMC4437063

[53] Somerville S, Dedman K, Hagan R, Oxnam E, Wettinger M, Byrne S, et al. The perinatal anxiety screening scale: development and preliminary validation. Archives of women’s mental health. 2014;17(5):443–54. doi: 10.1007/s00737-014-0425-8 24699796

[54] Brockington IF, Fraser C, Wilson D. The postpartum bonding questionnaire: a validation. Archives of Women’s Mental Health. 2006;9(5):233–42. doi: 10.1007/s00737-006-0132-1 16673041

[55] Higgins JPT, Thomas J, Chandler J, Cumpston M, Li T, Page MJ, et al, editor(s). *Cochrane Handbook for Systematic Reviews of Interventions* version 6.5 (updated August 2024). Cochrane, 2024. Available from www.cochrane.org/handbook

