# Supplementary material for: Effects of music medicine on anxiety among primigravida mothers of preterm neonates: The CalM MOM randomised controlled trial protocol
Source: PLoS One. 2026 Nov 3;21(11):e0360324. doi: 10.1371/journal.pone.0360324 (PMC13730379; doi:10.1371/journal.pone.0360324)
Supplement: Supplementary file 2 [file pone.0360324.s002.docx]

**Supplemental File 2:** **Estimand framework for the primary objective**

**A. Treatment**

- **Music Medicine intervention**: Classical South Indian instrumental music (*Raga Kalyani* and *Hindolam* played on the *Saraswati Veena* for 30 minutes daily) with Usual Care.
- **Nature Sounds comparator**: Nature sounds (sound of waves, river stream, and forest) for 30 minutes with Usual Care
- **Control**: Usual Care only.

**B. Population**

- Primigravida mothers with anxiety (screened using the Perinatal Anxiety Screening Scale [PASS]), whose preterm neonates (30-36 weeks gestation) are admitted to the neonatal intensive care unit (NICU) at Government Lady Goschen Hospital, Mangalore.
- **Inclusion criteria**: Mothers of preterm infants born via normal vaginal delivery, able to understand English or Kannada, with anxiety per PASS.
- **Exclusion criteria**: Mothers with known psychiatric illness, severe hearing loss, postpartum complications requiring intensive care, preterm twins, neonates with major congenital malformations or on invasive ventilatory support, prior experience with yoga or relaxation techniques, or professional singers/musicians.
- All participants will be randomized to Music Medicine with Usual Care, Nature Sounds with Usual Care, or Usual Care only groups.

**C. Variable of interest**

- Change in maternal State-Trait Anxiety Inventory (STAI) state anxiety score from baseline (Day 1, pre-intervention) to Day 7 (post-intervention).

**D. Intercurrent events and handling strategies**

Intercurrent events are events occurring after randomization that may affect the interpretation or existence of the outcome. The strategies align with the intention-to-treat principle where applicable, as implied by the study’s methodology.

1. **Withdrawal from the study**
   - **Description**: Participants may withdraw at any time, as stated in the informed consent.
   - **Strategy**: *Treatment Policy Strategy with Adjustment for Missing Data*
     - All randomized participants are included in the analysis.
     - Missing Day 7 STAI scores are handled using multiple imputation, assuming data are Missing at Random (MAR). This estimates the outcome as if the participant had continued, based on observed data trends.
2. **Non-adherence to the intervention/comparator**
   - **Description**: Participants in Music Medicine and Nature Sounds groups may not listen to the full 30-minute music/nature sounds session daily.
   - **Strategy**: *Treatment Policy Strategy*
     - Participants are analyzed according to their randomized group (Music Medicine, Nature Sounds, or control), regardless of adherence level. The effect is estimated as if all followed the protocol fully, reflecting real-world effectiveness.
3. **Use of additional relaxation techniques**
   - **Description**: Participants might use relaxation methods (e.g., meditation) during the study, though prior experience is an exclusion criterion.
   - **Strategy**: *Treatment Policy Strategy*
     - Such events are recorded but not adjusted for in the primary analysis. The effect is estimated irrespective of additional techniques, assuming randomization balances their occurrence.
4. **Changes in the neonate**’**s health status**
   - **Description**: Improvements or deteriorations in the neonate’s condition could influence maternal anxiety.
   - **Strategy**: *Treatment Policy Strategy*
     - These changes are monitored but not directly accounted for in the primary analysis. Randomization is assumed to distribute such events evenly across groups.
5. **Adverse events leading to discontinuation**
   - **Description**: Adverse events (e.g., maternal health complications) might prevent participation or lead to withdrawal.
   - **Strategy**: *Treatment Policy Strategy with Adjustment for Missing Data*
     - If discontinuation occurs, participants remain in the analysis per their randomized group, with missing STAI scores imputed using multiple imputation (MAR assumption). If no withdrawal occurs, the event is ignored.

**E. Population-level summary**

- The difference in the mean change in STAI state anxiety scores from baseline (Day 1, pre-intervention) to Day 7 (post-intervention) between the Music Medicine group, the Nature Sounds group, and the Usual Care group.
