# Supplementary material for: Effects of music medicine on anxiety among primigravida mothers of preterm neonates: The CalM MOM randomised controlled trial protocol
Source: PLoS One. 2026 Nov 3;21(11):e0360324. doi: 10.1371/journal.pone.0360324 (PMC13730379; doi:10.1371/journal.pone.0360324)
Supplement: Supplementary file 3 [file pone.0360324.s003.docx]

**Supplemental File 3**

**Supplemental File 3: Planned strategies to promote adequate enrolment**

To ensure sufficient recruitment and achieve the target sample size, the following strategies will be employed:

- **Systematic identification via patient registers**
  - Eligible participants will be identified using the patient registers of the NICU.
  - Recruitment will focus on mothers on postnatal day 2, when they are more likely to be physically stable and emotionally receptive.
- **Dedicated research staff involvement**
  - The RA and independent hospital staff member will be trained in empathetic communication and addressing participant concerns.
- **Reducing participant burden**
  - Music Medicine, delivered at 30 minutes per day for 7 days, will be conducted during times convenient for the mother while she is in the hospital, without requiring additional hospital visits or disrupting her routine.
  - The use of culturally familiar music and nature sounds is expected to enhance participant acceptability.
- **Recruitment monitoring and follow-up:**
  - A recruitment log will be maintained to document participant screening, consent status, and reasons for non-participation and exclusion.

——————————————————————————————————————-
