# Supplementary material for: Effects of music medicine on anxiety among primigravida mothers of preterm neonates: The CalM MOM randomised controlled trial protocol
Source: PLoS One. 2026 Nov 3;21(11):e0360324. doi: 10.1371/journal.pone.0360324 (PMC13730379; doi:10.1371/journal.pone.0360324)
Supplement: Supplementary file 4 [file pone.0360324.s004.docx]

**Supplemental File 4: Music-based interventions Checklist**

Supplemental File 4a: Reporting Guidelines for Music-based Interventions Checklist 2025

18

18

18-19

20

19-20

20

20

21

21

21

18

20

Supplemental File 4b: Pointers specific to classical Indian music 2025

| **Pointers** | **Page Number** |
| --- | --- |
| Has the participant undergone any training in CIM in the past?  If so, please specify the duration and level of training | No (Exclusion criteria) |
| Is this the participant's first time listening to CIM? | To be filled later |
| **Information on CIM** | |
| CIM Tradition  (North Indian (*Hindustani*)/South Indian (*Carnatic*) | 18 |
| Name of the *Raga* or *Ragas* | 19 |
| Person selecting the *Raga* (Patient/Investigator/Musician) | 18 |
| Rational for choosing this specific CIM | 18 |
| The emotion evoked by the *Raga* as described in traditional CIM texts |  |
| Provide the citation |  |
| Title of the composition | Non-lyrical |
| Is this vocal music or instrumental music? | 19  (Instrumental) |
| If instrumental, specify the musical instrument | 19 (*Saraswati Veena*) |
| Name of the vocalist/instrumentalist | 19 |
| Is there any drone accompaniment to the music?*(Eg:Tanpura, Swarmandal)* | 19, *Tanpura* |
| Are there any accompanying/supporting instruments? (Eg: *Mridangam, Ghatam, Morsing*, Flute, *Tabla*, Violin) | None |
| *Sruti* (Musical pitch) (Eg: 5.5 (G#) or 2.5 (D#) | 19 (D#) |
| Category of composition (Eg: *Keerthanam/Bandish/Padam/Thillana*) | Table 2a (*Raga alaapana* and *Taana*) |
| Name of the *Tala*/rhythmic cycle | NA |
| Describe the structure of the composition  (Eg: *Raga Alaapan/Taanam/Kalpanaswaram/Taniyavarthanam*) | Table 2a |
| Name of the composer | 19 |
| Language of the composition (Eg: Telugu/Tamil/Urudu/Hindi/Kannada/Malayalam) | NA |
| Meaning of the composition | NA |
| Is the participant able to understand the meaning of this music? | NA |

*Rajendran T, Patil A, Mohanta SK, Krishnadasa SN, Natarajan V. Classical Indian music for managing anxiety and pain among patients in a hospital setting: a systematic review and meta-analysis of randomized controlled trials. J Public Health (Oxf). 2025 Aug 29;47(3):527-539. doi: 10.1093/pubmed/fdaf048. PMID: 40331588.* <https://academic.oup.com/jpubhealth/article-abstract/47/3/527/8126303> By permission of Oxford University Press.
