# Supplementary material for: Effects of music medicine on anxiety among primigravida mothers of preterm neonates: The CalM MOM randomised controlled trial protocol
Source: PLoS One. 2026 Nov 3;21(11):e0360324. doi: 10.1371/journal.pone.0360324 (PMC13730379; doi:10.1371/journal.pone.0360324)
Supplement: Supplementary file 5 [file pone.0360324.s005.docx]

**Supplemental File 5**

**Supplemental file 5a: Harms-definition**

This is a **non-invasive, non-pharmacologic, low-risk behavioral intervention** involving passive listening to pre-recorded music or nature sounds. No physical or pharmacological exposure is involved. Based on prior literature, **no serious or life-threatening adverse events are expected**.

| **Category** | **Details** |
| --- | --- |
| **Expected Risk Level** | Less than minimal risk; non-invasive intervention; no physical or pharmacological exposure |
| **Systematically Assessed Harms** | Emotional discomfort, increased anxiety, abnormal vital signs |
| **Measurement Tools** | Pre- and post-intervention vital signs (BP, HR, RR)  Increased anxiety (self-reported questionnaires STAI/PASS)  Verbal self-report of discomfort **(Supplemental file 5b: Harms checklist – Daily session monitoring)** |
| **Assessment Time Points** | Day 1- Day 7 (pre- and post-session) |
| **Assessor** | Trained Research Assistant |
| **Metric and Aggregation** | Any observed or reported harm will be documented, including severity, timing, and resolution. |
| **Non-Systematically Assessed Harms** | Any spontaneously reported physical or emotional discomfort during the 7-day study period |
| **Collection Method** | Open-ended verbal feedback daily and during Day 7 debrief |
| **Assessment Period** | Day 1 (enrollment) to Day 7 (post-intervention close-out) |
| **Coding System** | *[WHO-UMC causality assessment system](https://www.who.int/docs/default-source/medicines/pharmacovigilance/whocausality-assessment.pdf)* |
| **Severity Grading** | Mild / Moderate / Severe; graded by PI or faculty supervisor |
| **Grouping of Harms** | By severity (mild, moderate, severe), causality (related, possibly related, unrelated), and whether it led to discontinuation |
| **Reporting of Adverse Events** | All significant or unexpected harms will be reported to:   - The **Institutional Ethics Committee (IEC)** - The **Trial Management Group (TMG)** |
| **Action on Harms** | Intervention paused or discontinued based on participant request or adverse reaction |

**——————————————————————————————————————-**

**Supplemental file 5b: Harms checklist – Daily session monitoring**

**Participant Number**: ____| **Group** (Blinded): □ A □ B □ C

**Date**: _____________ | **Day:**————————- **Time:**——————-

**Session #**: ___

**A. Pre-Session Monitoring** (Before Intervention)

| **Parameter** | **Value / Response** | **Notes (if any)** |
| --- | --- | --- |
| **Any signs of distress?**  (Refer:Monitoring charts+PASS/STAI) | □ Yes □ No | If yes, describe |
| **Participant appears willing to proceed?** | □ Yes □ No |  |

**B. During Session** (Participant will be alone in the room, but participant can notify the staff and leave the room at any moment if they feel uncomfortable)

| **Observation Area** | **Response** | **Notes (if any)** |
| --- | --- | --- |
| **Any interruption or early stop?** | □ Yes □ No | If yes, reason |

**C. Post-Session Monitoring** (Immediately After Intervention)

| **Parameter** | **Value / Response** | **Notes (if any)** |
| --- | --- | --- |
| **Participant reported feeling:** | □ Relaxed □ Neutral □ Anxious □ Other: _______ |  |
| **Any adverse effects reported?** | □ Yes □ No | If yes, describe |
| **Participant willing to continue?** | □ Yes □ No |  |

**D. Summary & RA Signature**

- **Any harm reported or observed?** □ Yes	□ No
- **Details (if applicable):** ___________________________________________________________
- **Action Taken (if any):** ____________________________________________________________
- **RA Name & Signature:** _______________________
- **Notified PI?** □ Yes □ No
- **Notified the faculty supervisor?** □ Yes □ No
