# Supplementary material for: Effects of music medicine on anxiety among primigravida mothers of preterm neonates: The CalM MOM randomised controlled trial protocol
Source: PLoS One. 2026 Nov 3;21(11):e0360324. doi: 10.1371/journal.pone.0360324 (PMC13730379; doi:10.1371/journal.pone.0360324)
Supplement: Supplementary file 6 [file pone.0360324.s006.docx]

**Supplemental File 6**

| **Supplemental file 6a: Monitoring chart-Music Medicine/Nature Sounds arms** | | | | | | | | | | | | | | |
| --- | --- | --- | --- | --- | --- | --- | --- | --- | --- | --- | --- | --- | --- | --- |
| **GROUP NUMBER: PARTICIPANT NUMBER: Anxiety pre-enrollment Screening (PASS scores):** | | | | | | | | | | | | | | |
| **POST NATAL DAY** | **PND 2** | | **PND 3** | | **PND 4** | | **PND 5** | | **PND 6** | | **PND 7** | | **PND 8** | |
| **INTERVENTION DAY** | **DAY 1** | | **DAY 2** | | **DAY 3** | | **DAY 4** | | **DAY 5** | | **DAY 6** | | **DAY 7** | |
| **DATE AND DAY** |  | |  | |  | |  | |  | |  | |  | |
| **TIME OF INTERVENTION (Eg:3pm)** |  | |  | |  | |  | |  | |  | |  | |
| **DECIBEL LEVEL** |  | |  | |  | |  | |  | |  | |  | |
| **DISTANCE BETWEEN SPEAKER & PARTICIPANT** |  | |  | |  | |  | |  | |  | |  | |
| **MEASUREMENT PHASE** | **Pre-I** | Post-I | **Pre-I** | Post-I | **Pre-I** | Post-I | **Pre-I** | Post-I | **Pre-I** | Post-I | **Pre-I** | Post-I | **Pre-I** | Post-I |
| **ANXIETY (STAI SCORES)** |  |  | NA | NA | NA | NA | NA | NA | NA | NA | NA | NA |  |  |
| **ANXIETY (PASS SCORES)** |  |  | NA | NA |  |  | NA | NA |  |  | NA | NA |  |  |
| **Postpartum Bonding Questionnaire (PBQ)** |  |  |  |  |  |  |  |  |  |  |  |  |  |  |
| **BLOOD PRESSURE** |  |  |  |  |  |  |  |  |  |  |  |  |  |  |
| **PULSE RATE** |  |  |  |  |  |  |  |  |  |  |  |  |  |  |
| **RESPIRATORY RATE** |  |  |  |  |  |  |  |  |  |  |  |  |  |  |
| **BREAST MILK QUANTITY** | NA | |  | | NA | | NA | | NA | | NA | |  | |
| **NEONATAL WEIGHT** |  | | NA | | NA | | NA | | NA | | NA | |  | |
| **Neonatal condition** |  | |  | |  | |  | |  | |  | |  | |
| **ANY HARM (Distress)**  **(If none, specify ‘None’)** |  | |  | |  | |  | |  | |  | |  | |
| **USUAL CARE** |  | |  | |  | |  | |  | |  | |  | |

| **Supplemental file 6b: Monitoring chart-control arm** | | | | | | | |
| --- | --- | --- | --- | --- | --- | --- | --- |
| **GROUP NUMBER: PARTICIPANT NUMBER: Anxiety pre-enrollment (PASS scores):** | | | | | | | |
| **POST NATAL DAY** | **PND 2** | **PND 3** | **PND 4** | **PND 5** | **PND 6** | **PND 7** | **PND 8** |
| **INTERVENTION DAY** | **DAY 1** | **DAY 2** | **DAY 3** | **DAY 4** | **DAY 5** | **DAY 6** | **DAY 7** |
| **DATE AND DAY** |  |  |  |  |  |  |  |
| **ANXIETY (STAI SCORES)** |  | NA | NA | NA | NA | NA |  |
| **ANXIETY (PASS SCORES)** |  | NA |  | NA |  | NA |  |
| **Postpartum Bonding Questionnaire (PBQ)** |  |  |  |  |  |  |  |
| **BLOOD PRESSURE** |  |  |  |  |  |  |  |
| **PULSE RATE** |  |  |  |  |  |  |  |
| **RESPIRATORY RATE** |  |  |  |  |  |  |  |
| **BREAST MILK QUANTITY** | NA |  | NA | NA | NA | NA |  |
| **NEONATAL WEIGHT** |  | NA | NA | NA | NA | NA |  |
| **Neonatal condition** |  |  |  |  |  |  |  |
| **USUAL CARE** |  |  |  |  |  |  |  |

NA=Not Applicable
