# Supplementary material for: Effects of music medicine on anxiety among primigravida mothers of preterm neonates: The CalM MOM randomised controlled trial protocol
Source: PLoS One. 2026 Nov 3;21(11):e0360324. doi: 10.1371/journal.pone.0360324 (PMC13730379; doi:10.1371/journal.pone.0360324)
Supplement: Supplementary file 7 [file pone.0360324.s007.docx]

**Supplemental File 7: Investigator Roles and Responsibilities**

**Roles and responsibilities**

| **NAMES, AFFILIATIONS, AND ROLES OF PROTOCOL CONTRIBUTORS** | | |
| --- | --- | --- |
| **Name and Role** | **Affiliation** | **Contributions** |
| **Dr. Bishnupriya Mukherjee, MBBS**  **Principal Investigator** | Department of Pediatrics, Kasturba Medical College, Mangalore, Manipal Academy of Higher Education, India | Led the overall study, conceptualisation the RCT design, presented at the regulatory meetings, developed the methodology, designed and led the Perinatal Anxiety Screening Scale (PASS)-*Kannada* validity study and developed the master data chart, and coordinated departmental and institutional support for the project.  Drafted the protocol.  Approved the final protocol |
| **Dr. Tara Rajendran, MBBS, Ph.D., MFA**  **Co-Principal Investigator** | Department of Music, Faculty of Fine Arts, Annamalai University, Annamalai Nagar, Chidambaram, Tamil Nadu, India | Conceptualized and designed the RCT, developed the methodology, led the literature review, designed the interventions/comparators, and composed and recorded the music-based intervention.  Led the protocol drafting.  Approved the final protocol |
| **Anjali Maria Augustine** | Department of Psychology, York University, Toronto, Canada | Led the Perinatal Anxiety Screening Scale (PASS)-*Kannada* validity study, developed the master data chart, associated data entry processes, and structured data collection sheet for the RCT.  Drafted the protocol.  Approved the final protocol |
| **Akshay Patil, MSc**  **Independent statistician** | University Hospitals of Derby and Burton NHS Foundation Trust, Uttoxeter Road, Derby, DE22 3NE, United Kingdom | Led the statistical design for the RCT and drafted the statistical part of the protocol.  Approved the final protocol |
| **Subham Kumar Mohanta, MPhil**  **Study statistician** | Global Biometrics and Data Sciences, Bristol Myers Squibb, Madhapur, Hyderabad, Telengana, India | Co-led the statistical design for the RCT and drafted the statistical part of the protocol.  Approved the final protocol |
| **Dr. Nutan Kamath, MD**  **Faculty Supervisor, Clinician** | Department of Pediatrics, Kasturba Medical College, Mangalore, Manipal Academy of Higher Education, India  [nutan.kamath@manipal.edu](mailto:nutan.kamath@manipal.edu) | Conceptualized the study design, developed the methodology, provided clinical oversight and mentorship throughout the study design and protocol refinement; offered critical review and guidance on feasibility and implementation strategies.  Edited the protocol.  Approved the final protocol |
