# Supplementary material for: Effects of music medicine on anxiety among primigravida mothers of preterm neonates: The CalM MOM randomised controlled trial protocol
Source: PLoS One. 2026 Nov 3;21(11):e0360324. doi: 10.1371/journal.pone.0360324 (PMC13730379; doi:10.1371/journal.pone.0360324)
Supplement: Supplementary file 8 [file pone.0360324.s008.docx]

**STUDY MONITORING GROUPS**

**Data monitoring committee (DMC)**

A formal DMC will not be established for this study due to the less-than-minimal risk nature of the intervention; this is a non-invasive intervention trial involving auditory stimuli (Music Medicine and Nature Sounds), and no pharmacological or high-risk interventions are involved. No significant adverse events are anticipated. Participant safety will be ensured throughout the study. Those who directly interact with the patients will be trained in research ethics and [Good Clinical Practice (GCP)](https://database.ich.org/sites/default/files/ICH_E6-R3_GCP-Principles_Draft_2021_0419.pdf) principles. Should any unexpected events or concerns arise, they will be immediately reported to the IEC, and appropriate corrective action will be taken.

**Trial monitoring**

Trial conduct will be monitored internally on a weekly basis. A study log and a data collection checklist will be maintained.

Monitoring activities will include:

- Ensuring protocol adherence
- Verifying informed consent procedures
- Maintaining confidentiality protocols

Given the limited scope, short duration (7-day intervention), and less than minimal risk involved, no external or independent trial monitor is planned.

**Trial Management Group (TMG)**

The TMG is responsible for the day-to-day operational management of the trial, including recruitment, intervention delivery, data collection, and safety monitoring.

| **Role** | **Suggested Member** | **Responsibility** |
| --- | --- | --- |
| Principal Investigator (Clinical) | Dr. Bishnupriya Mukherjee | Overall trial leadership, clinical oversight and participant safety |
| Co-Principal Investigator (Music) | Dr. Tara Rajendran | Music intervention fidelity |
| Study Statistician | Shubham Kumar Mohanta | Statistical planning and data quality |
| Research Assistant (RA) | To be nominated | Recruitment, data collection, and logistics |
| Faculty Supervisor (Chair) | Dr. Nutan Kamath | Final decisions |

**Trial Steering Committee (TSC)**

The TSC provides independent oversight of the trial. It reviews progress, approves major decisions (such as sample size adjustments following the Blinded Sample Size Re-estimation), and ensures the scientific and ethical integrity of the study.

| **Position** | **Suggested Profile** | **Status** | **Role** |
| --- | --- | --- | --- |
| **Independent Chair** | Senior academic/clinician (external to study setting) | Independent | Leads TSC meetings and provides strategic oversight |
| **Independent Clinicians** | Neonatologist, Obstetrician, and a Psychiatrist (external) | Independent | Clinical and safety oversight |
| **Independent Statistician** | Akshay Patil | Fully Independent | Reviews statistical methods and BSSR reports |
| **Lay/Patient Representative** | Mother with experience of preterm birth | Independent | Patient and public perspective |
| **Principal Investigator** | Dr. Bishnupriya Mukherjee | Non-independent | Reports progress (non-voting member) |
| **Study Statistician** | Shubham Kumar Mohanta | Non-independent | Provides updates (non-voting member) |

**Relationship to funder:** Independent (No funders yet).

**Data Management Team (DMT)**
Handles secure data storage, quality assurance, database maintenance, and preparation for statistical analysis.
**Membership:** Research Assistant, independent hospital staff member, and oversight by PI and faculty supervisor.

**Endpoint Adjudication Committee**
Given the less-than-minimal-risk nature of the music intervention and use of validated self-reported outcomes, an Endpoint Adjudication Committee is not required.
