# Supplementary material for: Effects of music medicine on anxiety among primigravida mothers of preterm neonates: The CalM MOM randomised controlled trial protocol
Source: PLoS One. 2026 Nov 3;21(11):e0360324. doi: 10.1371/journal.pone.0360324 (PMC13730379; doi:10.1371/journal.pone.0360324)
Supplement: Supplementary file 9 [file pone.0360324.s009.docx]

**Supplemental File 9: Additional Details**

**ETHICS**

**Protocol amendments**

Any significant changes to the study protocol, including modifications to the study design, eligibility criteria, intervention procedures, outcome measures, consent process, or data management, will be promptly reported to the IEC for review and approval before implementation.

Upon IEC approval, the following steps will be taken:

- All study team members will be informed through written communication and a team meeting.
- Updated documents will be circulated and archived appropriately.
- Trial registry (CTRI) will be updated if applicable.
- Any modifications that may affect participant safety or study validity will be clearly explained to participants, and re-consent will be obtained if necessary.

All protocol amendments and version changes will be documented with version numbers and dates

**Written** **informed consent process**

**Role and experience of the individual obtaining consent:**

Written informed consent will be obtained, and before initiating the study, the personnel obtaining consent will undergo a 1-week structured training program conducted by the PI. The training will include:

•	Ethical principles of human subject research

•	Study-specific procedures

•	Use of a checklist

•	Role-playing and simulation of the consent process practice sessions

•	Privacy and confidentiality protocols

Practice sessions will be supervised by the PI to ensure consistency and adherence to ethical standards.

**Consent Process**

•	Each eligible participant will be approached in a private and non-coercive environment.

•	A Participant Information Sheet (PIS) in the local language will be provided and explained verbally. This includes details on study purpose, procedures, risks, benefits, voluntary participation, withdrawal rights, confidentiality, and some frequently asked questions.

•	Adequate time will be given for questions and consideration.

•	Written Informed Consent will be obtained, with a copy provided to the participant, if asked.

**CONFIDENTIALITY**

**1. Collection and Storage of Personal Information:**

•	All personal and health-related information collected during the study will be securely stored in locked cabinets (for physical forms) and password-protected digital files (for electronic data).

•	Each participant will be assigned a participant number to anonymize their data.

**2. During the Trial:**

•	Data collection forms will use coded identifiers without names or contact details.

•	All electronic devices used for data entry will be password-protected, and access will be restricted to authorized personnel.

**3. After the Trial:**

•	Identifiable information will be retained for a defined period (as per IEC/Indian Council of Medical Research guidelines) and then securely destroyed.

**4. Data Sharing with Co-Investigators:**

•	Before sharing with co-investigators, all personal identifiers will be removed (name, hospital ID, contact details, etc.).

•	Only de-identified datasets containing study codes will be transmitted.

•	Data will be transferred using secure, encrypted channels

**ANCILLARY AND POST-TRIAL CARE**

**1. Ancillary Care During the Trial:**

Participants who are identified as having elevated anxiety levels based on the validated questionnaires will be referred to a licensed clinical psychologist for further evaluation and management. This referral will be facilitated by the PI in collaboration with the Department of Pediatrics.

**2. Post-trial care and benefits:**

At the conclusion of the study, all participants will be provided with a complimentary digital album of South Indian classical *Saraswati Veena* music, regardless of group allocation. This is intended as a token of appreciation and a potential resource for continued stress reduction. No other post-trial medical benefits are planned.

**3. Compensation for trial-related harm:**

No physical or psychological harm is expected from participation in this study, as the intervention involves listening to soothing audio.

**PATIENT AND PUBLIC INVOLVEMENT**

Patient and public involvement is ensured through the inclusion of a lay representative, a mother with lived experience of preterm birth, as an independent member of the Trial Steering Committee, contributing a patient and public perspective to trial oversight.

**DISSEMINATION POLICY**

The results will be submitted for publication in peer-reviewed journals and presented at relevant national and international conferences, particularly those focused on pediatrics and perinatal mental health. Authorship will follow the International Committee of Medical Journal Editors (ICMJE) criteria.

**DATA SHARING**

**1. Data Availability and Application Process:**

De-identified individual participant data, along with the study protocol, statistical analysis plan, and informed consent form, will be made available upon reasonable request. Researchers interested in accessing the data should submit a written proposal detailing the intended use, objectives, and data handling procedures. Proposals will be reviewed by the PI, faculty supervisor, and the IEC at Kasturba Medical College, Mangalore.

**2. Data Transfer Process:**

Approved applicants will be required to sign a data use agreement (DUA) outlining confidentiality, data protection, and guidelines. Data will be transferred through a secure, encrypted platform (e.g., institutional email with password-protected files, secure cloud sharing platform) to ensure privacy and data integrity.

**3. Informed Consent:**

Participants are assured that no personal identifiers will be shared, and their privacy will be strictly protected, and this is included in the informed consent.

**4. Restrictions and Governance:**

Data will not be used for commercial purposes. Any secondary analyses must be approved by the faculty supervisor and require additional IEC clearance. The data will be available for five years after the study publication.

5. **Individual trial participant data sharing statement**

De-identified individual participant data that underlie the results reported in this article will be made available to researchers upon reasonable request to the corresponding author/faculty supervisor, following publication of the study results. Data will be shared for academic research purposes, and requests will be reviewed on a case-by-case basis. Proposals will be reviewed by the faculty supervisor and the Institutional Ethics Committee (IEC) at Kasturba Medical College, Mangalore. A data sharing agreement may be required.

**Artificial intelligence was not used for the preparation of this protocol.**
