# Supplementary material for: Effects of music medicine on anxiety among primigravida mothers of preterm neonates: The CalM MOM randomised controlled trial protocol
Source: PLoS One. 2026 Nov 3;21(11):e0360324. doi: 10.1371/journal.pone.0360324 (PMC13730379; doi:10.1371/journal.pone.0360324)
Supplement: Supplementary file 10 [file pone.0360324.s011.docx]

**Response to Reviewers**

Manuscript: **PONE-D-25-68653**

*Effects of music medicine on anxiety among primigravida mothers of preterm neonates: The CalM MOM randomised controlled trial protocol*

We sincerely thank the Editor and both the Reviewers for their thoughtful comments and the opportunity to revise our manuscript. We greatly appreciate the time and effort invested in reviewing our work. We have carefully addressed all comments, and the revisions have substantially improved the quality and clarity of the manuscript! The review process has also been a valuable learning experience for us, and we are grateful for your constructive feedback!

|  | **Changes to be Made** | **Response from Authors** |
| --- | --- | --- |
|  | **Academic Editor Comments (Prof. Sascha Köpke)** | |
| 1 | AE1. **Abstract**: The abstract is definitely the weakest part of this protocol, please out more effort in it to optimize the paper. The abstract should be **shortened and restructured.** Please be **more concise and avoid repetitions** e.g. on the trial being the first in India. Therefore, most of the text at the end of the abstract starting with “This is the first RCT from India…” can be **deleted**, including the mention of the **UNSDGs**. On the other hand, important information e.g. concerning **randomization and allocation concealment** is missing. Please check e.g. the CONSORT guideline for abstracts. | We thank the Academic Editor for this comment. The abstract has been substantially restructured. And w**e have removed the repetitive sentences** beginning *“This is the first RCT from India…”* and the reference to UN SDGs.  The following mandatory CONSORT 2025 abstract elements have been added or clarified:  ✅**Randomisation:**✅**Allocation concealment**✅**Blinding**✅**Revised** **Sample size**  **Background:** Preterm birth and Neonatal Intensive Care Unit (NICU) admission are significant stressors that heighten maternal anxiety. Although RCTs have reported that music-based interventions used alongside usual care (UC) are effective and low-cost in reducing maternal anxiety, evidence from India remains limited.  **Objective:** Primary objective is to assess the impact of listening to recorded classical Indian Instrumental *Saraswati Veena* music (*Ragas Kalyani* and *Hindolam*) (30-minutes/day over 7-days) alongside UC on maternal anxiety (*STAI-state anxiety)* of primigravida women with preterm neonates in the NICU. Secondary objectives measure changes in anxiety (Perinatal Anxiety Screening Scale (PASS)), maternal-infant bonding, breast milk volume, and neonatal body weight.  **Method:** This three-arm parallel-group, superiority RCT among primigravida mothers with anxiety (screened using PASS) of preterm neonates (30-36 weeks of gestation, admitted to NICU) delivered vaginally at Government Lady Goschen Hospital, Mangalore. The intervention arm will receive Music Medicine (MM), the comparator arm Nature Sounds (NS), and the control arm UC. Participant blinding is not feasible (auditory intervention), and they serve as their own outcome-assessors (self-reported questionnaires); investigators and the independent statistician will remain blinded. Personnel interacting with participants are blinded to study objectives; enrolment/allocation staff blinded to the randomization sequence, and assessment staff blinded to group identity.  Randomization will employ a STATA-generated, permuted stratified block design stratified by baseline anxiety severity and gestational age (Allocation concealment: Sequentially Numbered, Opaque, Sealed Envelopes; Allocation Ratio: 1:1:1). Sample size (66/arm; total N=198) was determined through a simulation-based power analysis (5,000 iterations, base-R), assuming an MCID of 4 (MM) and 2 (NS) on the STAI, residual SD of ~8, powered at 80% (α=0.05). Blinded sample-size re-estimation will be conducted at 50% enrollment completion. Primary powered analysis will employ ANCOVA (baseline-adjusted), and Secondary (exploratory) analyses will utilize a linear mixed-effects model for longitudinal continuous data with time × group interaction. |
| 2 | AE2a. Methods (a): How will you avoid contamination between groups? | Thanks so much for raising this important point! We have added **‘Strategies to Minimise Contamination Between Study Arms’ (Line 445-466) under Intervention details’.**  ***Strategies to minimise contamination between study arms***  *As the study will be conducted in a resource-limited setting, dedicated facilities to accommodate participants from different study arms in separate rooms will not be available, and mothers will continue to reside in the same postnatal wards. However, to minimise contamination between the three arms, the following strategies will be implemented:*   - ***Private study setting:*** *All 30-minute intervention sessions (Music Medicine and Nature Sounds) will be conducted in a designated quiet private room separate from the shared wards. Participants in the usual care arm will receive no audio intervention.* - ***Individual staff supervision:*** *An independent hospital staff member, masked to the study objectives, will escort each participant to the intervention room, activate only the arm-specific audio file on the laptop, and then leave the room. The RA will conduct pre- and post-session assessments but will not be present during intervention delivery.* - ***Sequential scheduling:*** *As participants from different study arms reside in the same postnatal wards, intervention sessions will be individually scheduled and conducted sequentially to minimise contact between participants undergoing different interventions. Real-time coordination will be maintained through recruitment and intervention logs.* - ***Participant instructions:*** *During the informed consent process, participants will be explicitly instructed not to discuss the content or details of their sessions with other mothers in the ward.* - ***Fidelity documentation:*** *Monitoring charts (Supplementary File 7) will record the exact session time, decibel level, and speaker-to-participant distance for every intervention session to ensure protocol adherence.* |
| 3 | AE2b. Methods (b): L. 151: “Stratified random sampling” is mentioned (l. 151), but strata are not reported. | Thank you for pointing this out. In Line 158 we have clarified this as *‘stratified on baseline anxiety severity and gestational age (dichotomised).’* |
| 4 | AE2c. Methods (c): L. 317: “professors cum professional musicians”, do you mean “with”? | Thank you for pointing this out! Yes! They are both professors and professional musicians. I have replaced ‘cum’ with ‘and’. |
| 5 | AE2d. Methods (d) L. 465: “Mothers will be screened from the nurse’s record book.” What is meant be screening here? Is this really about “Data collection“? | We thank the editor for pointing out this ambiguity. We agree the sentence was redundant with the eligibility screening process already described in the same paragraph and have removed it to avoid confusion between recruitment/screening and data collection. |
| 6 | AE3a Discussion: (a) What is the probability of the mothers staying in hospital for all 7 days? Is this common? Could this be a problem? | As per the Facility-Based Newborn Care guidelines of the Ministry of Health and Family Welfare, Government of India, preterm infants are discharged only after achieving clinical stability, establishing adequate feeding, and demonstrating sustained weight gain. In our setting, it is common for preterm neonates to remain hospitalized for at least two weeks, until they reach an appropriate discharge weight (typically around 1.8 kg), are off all antibiotics, are taking full oral feeds, and the mother is confident in caring for her infant. Therefore, the probability of mothers remaining available for the full 7-day intervention period is expected to be high. We do not anticipate substantial attrition due to early neonatal discharge; however, any unexpected maternal or neonatal discharge will be documented and accounted for in the analysis. : <https://www.iapsm.org/pdf/Guidelines/RMNCH%2BA/Guidelines%20for%20Facility%20Based%20Newborn%20Care%202011.pdf> |
| 7 | AE3b. Please discuss why there is no patient and/or public involvement in this study. | We thank the reviewer for encouraging us to consider this, which will improve the trial. Patient and public involvement is incorporated via a Lay/Patient Representative (a mother with lived experience of preterm birth) serving as an independent member of the Trial Steering Committee, providing patient perspective on trial oversight and major decisions. This has been clarified in **Supplemental File 8.** |
|  | **Journal Requirements** | |
| 1 | JR1. Please ensure that your manuscript meets PLOS ONE's style requirements, including those for file naming. The PLOS ONE style templates can be found at <https://journals.plos.org/plosone/s/file?id=wjVg/PLOSOne_formatting_sample_main_body.pdf> and <https://journals.plos.org/plosone/s/file?id=ba62/PLOSOne_formatting_sample_title_authors_affiliations.pdf> | Thank you! We have followed this style! |
| 2 | JR2. Please include your tables as part of your main manuscript and remove the individual files. Please note that supplementary tables (should remain/ be uploaded) as separate "supporting information" files. | Thank you! We have included the tables as part of the main manuscript and have removed the individual files. |
| 3 | JR3. We note that the original protocol that you have uploaded as a Supporting Information file contains an institutional logo. As this logo is likely copyrighted, we ask that you please remove it from this file and upload an updated version upon resubmission. | We have removed the MAHE Kasturba Medical College logo from the Supplemental File 9 |
| 4 | JR4. If the reviewer comments include a recommendation to cite specific previously published works, please review and evaluate these publications to determine whether they are relevant and should be cited. There is no requirement to cite these works unless the editor has indicated otherwise. | Not applicable. |
| 5 | JR5. Please review your reference list to ensure that it is complete and correct. If you have cited papers that have been retracted, please include the rationale for doing so in the manuscript text, or remove these references and replace them with relevant current references. Any changes to the reference list should be mentioned in the rebuttal letter that accompanies your revised manuscript. If you need to cite a retracted article, indicate the article’s retracted status in the References list and also include a citation and full reference for the retraction notice. | Not applicable. |
|  | **Reviewer #1 Comments** | |
|  | This is a very well written paper describing the protocol of a three arm parallel group, observer blinded, superiority randomized controlled  trial testing music based intervention for anxiety among mothers of preterm neonates. I have a few suggestions that may improve the future analysis of  the study. | Thanks very much for your kind comments! We really appreciate it! |
| 1 | R1-1. The sample size calculation is extremely thorough and well documented. However, the current calculation appears to support a pre post two group comparison of main effects under an ANCOVA framework as implemented in the pwr package. In particular, the function pwr.f2.test() assumes a standard linear model and does not account for within subject correlation that arises in repeated measures designs. For longitudinal studies, power estimates based on this approach can be optimistic and may not accurately reflect the required sample size. This issue would not arise if the design were strictly a pre-post comparison with a corresponding analysis strategy. However, in lines 495 to 500 there are indications that a linear mixed effects model will be used, including testing for a time by group interaction (also referenced in line 529). The power analysis presented earlier does not correspond to this type of model. For longitudinal designs, one could instead use the R package “longpower” with appropriate parameter specification to estimate power. Typically, the required sample size for detecting a time by group interaction in a three arm trial would be larger. | We thank the reviewer for this suggestion! We fully accept the point.  **Clarification: (Line 227)**  Simulation-based sample size estimation   - **Primary powered analysis:** ANCOVA on STAI change from baseline to Day 7 (adjusting for baseline). - **Secondary/exploratory:** A linear mixed-effects model for analysing longitudinal continuous data — with time × group interaction (no formal power estimation for secondary exploratory endpoints)   Sample Size Justification – Simulation-Based Power Analysis (Evidence in Governance and Politics (EGAP) methodology)  We conducted a transparent simulation-based power analysis (5,000 iterations) for the primary ANCOVA contrast (Music vs Nature sounds vs Control, Minimal Clinically Important Difference (MCID) = 4 points for Music Medicine, MCID=2 points for Nature Sounds, residual standard deviation (SD) ≈ 8, baseline adjustment).  **Complete R Code Used for Revised Power Calculation (Base R – EGAP style)**  The following self-contained base-R script was used to generate the power estimates above:  set.seed(123)  possible_n_per_arm <- seq(20, 100, by = 2)  powers <- rep(NA, length(possible_n_per_arm))  alpha <- 0.05; sims <- 5000  for (j in 1:length(possible_n_per_arm)) {    n_per_arm <- possible_n_per_arm[j]; N <- n_per_arm * 3    significant <- rep(NA, sims)    for (i in 1:sims) {      baseline <- rnorm(N, 45, 10)      Y0 <- baseline + rnorm(N, 0, 8)          # Control      Y_music <- Y0 - 4; Y_nature <- Y0 - 2   # Effects (no direct reference: Nature Sounds comparator would have roughly half the effect of the Music Medicine intervention, will be revised during SSR)      Z <- sample(c("Control","Music","Nature"), N, TRUE, c(1/3,1/3,1/3))      Y <- ifelse(Z=="Music", Y_music, ifelse(Z=="Nature", Y_nature, Y0))      fit <- lm(Y ~ Z + baseline)      p <- summary(fit)$coefficients["ZMusic", "Pr(>\|t\|)"]      significant[i] <- (p <= alpha)    }    powers[j] <- mean(significant)  }  data.frame(n_per_arm = possible_n_per_arm, power = round(powers, 3))  And Line 266 we have added a Blinded Sample Size Re-estimation (BSSR) Plan & Recruitment Forecast |
|  |  |  |
| 2 | R1-2. I suggest that the authors clearly specify in the “Secondary endpoint analysis” section which model corresponds to the powered primary hypothesis and which analyses are not explicitly powered and should therefore be considered secondary or exploratory. This clarification would help readers understand the distinction between the primary analysis and the additional longitudinal modeling described later in the manuscript. | Thanks very much for pointint this out! We really appreciate it! We have now revised the Statistical Analysis section with two clear subsections:  **Line: 227**  *Simulation-based sample size estimation*   - ***Primary powered analysis:*** *ANCOVA on STAI change from baseline to Day 7 (adjusting for baseline).* - ***Secondary/exploratory:*** *A linear mixed-effects model for analysing longitudinal continuous data — with time × group interaction (no formal power estimation for secondary exploratory endpoints)*   **Line 549**  ***Primary Endpoint Analysis***  *The primary outcome is the change in STAI state anxiety score from baseline (Day 1 pre-intervention) to Day 7 post-intervention.*  *The primary analysis will be an ANCOVA comparing the mean change in STAI scores between the three groups, adjusting for baseline STAI score. This analysis corresponds to the pre-specified powered primary hypothesis.*  ***Assumption checking and alternative analysis:*** *Prior to analysis, normality of residuals and homogeneity of variances will be assessed. If these assumptions are violated, the Van Elteren test (stratified Wilcoxon rank-sum test) will be used as the primary non-parametric analysis, stratified by baseline anxiety level and gestational age (dichotomised at the median or clinically relevant cut-off).*  *If the overall test is statistically significant, post-hoc pairwise comparisons will be performed using Bonferroni correction.*  ***Secondary Endpoint Analyses***  *All secondary endpoint analyses are considered exploratory. No adjustment for multiplicity will be applied.*   - ***Perinatal Anxiety Screening Scale****: A mixed-effects model for repeated measures (MMRM) or repeated measures ANCOVA will be used to compare trajectories over time (Days 1, 3, 5, and 7), adjusting for baseline score where appropriate. Group × time interaction will be explored.* - ***Postpartum Bonding Questionnaire****: ANCOVA or MMRM will be used to compare total and subscale scores, with adjustment for baseline score if relevant.* - ***Breast Milk Volume (Day 2 and Day 7)****: ANCOVA on Day 7 values adjusting for Day 2 measurement, or MMRM if appropriate.* - ***Neonatal Body Weight (Day 1 and Day 7)****: ANCOVA on Day 7 weight, adjusting for baseline weight and relevant covariates (e.g., gestational age at birth).* - ***Vital Parameters (Blood Pressure, Pulse Rate, Respiratory Rate)****: Within-group pre-post changes will be assessed using paired t-tests. Between-group comparisons at individual time points will use ANOVA or ANCOVA. Time-by-group interaction effects will be explored using MMRM. These analyses are exploratory.* |
|  | **Reviewer #2 Comments** | |
| 1 | R2-1. Dear Authors, Thank you for giving me the opportunity to review the article. It is recommended that you clarify whether the article tests a model or discusses research results, and discuss them by integrating them with the findings. | Thank you! This is a protocol of a randomised controlled trial. To clarify this we have written ‘Protocol’ in the title and we have used future tense throughout the manuscript. |
