# Supplementary material for: Effects of music medicine on anxiety among primigravida mothers of preterm neonates: The CalM MOM randomised controlled trial protocol
Source: PLoS One. 2026 Nov 3;21(11):e0360324. doi: 10.1371/journal.pone.0360324 (PMC13730379; doi:10.1371/journal.pone.0360324)
Supplement: Peer review history

# Peer review: Decision Letter 0

**DOI:** 10.1371/journal.pone.0360324.r001

**Authors:** Sascha Köpke

29 May 2026

Dear Dr. Kamath,

Thank you for submitting your manuscript to PLOS ONE. After careful consideration, we feel that it has merit but does not fully meet PLOS ONE’s publication criteria as it currently stands. Therefore, we invite you to submit a revised version of the manuscript that addresses the points raised during the review process.

Thank you for the well structured protocol. As the non-statistical review is very short, I have added some further comments:

(1) Abstract: The abstract is definitely the weakest part of this protocol, please out more effort in it to optimize the paper. The abstract should be shortened and restructured. Please be more concise and avoid repetitions e.g. on the trial being the first in India. Therefore, most of the text at the end of the abstract starting with “This is the first RCT from India…” can be deleted, including the mention of the UNSDGs. On the other hand, important information e.g. concerning randomization and allocation concealment is missing. Please check e.g. the CONSORT guideline for abstracts.

(2) Methods: (a) How will you avoid contamination between groups?; (b) L. 151: “Stratified random sampling” is mentioned (l. 151), but strata are not reported.; (c) L. 317: “professors cum professional musicians”, do you mean “with”?; (d) L. 465: “Mothers will be screened from the nurse’s record book.” What is meant be screening here? Is this really about “Data collection“?

(3) Discussion: (a) What is the probability of the mothers staying in hospital for all 7 days? Is this common? Could this be a problem?; (b) Please discuss why there is no patient and/or public involvement in this study.

Please submit your revised manuscript by Jul 13 2026 11:59PM. If you will need more time than this to complete your revisions, please reply to this message or contact the journal office at plosone@plos.org. When you're ready to submit your revision, log on to https://www.editorialmanager.com/pone/ and select the 'Submissions Needing Revision' folder to locate your manuscript file.

- A letter that responds to each point raised by the academic editor and reviewer(s). You should upload this letter as a separate file labeled 'Response to Reviewers'.
- A marked-up copy of your manuscript that highlights changes made to the original version. You should upload this as a separate file labeled 'Revised Manuscript with Track Changes'.
- An unmarked version of your revised paper without tracked changes. You should upload this as a separate file labeled 'Manuscript'.

If you would like to make changes to your financial disclosure, please include your updated statement in your cover letter. Guidelines for resubmitting your figure files are available below the reviewer comments at the end of this letter.

As the corresponding author, your ORCID iD is verified in the submission system and will appear in the published article. PLOS supports the use of ORCID, and we encourage all coauthors to register for an ORCID iD and use it as well. Please encourage your coauthors to verify their ORCID iD within the submission system before final acceptance, as unverified ORCID iDs will not appear in the published article. *Only*  the individual author can complete the verification step; PLOS staff *cannot*  verify ORCID iDs on behalf of authors.

We look forward to receiving your revised manuscript.

Kind regards,

Sascha Köpke

Academic Editor

PLOS One

**Journal Requirements:**

When submitting your revision, we need you to address these additional requirements.

1. Please ensure that your manuscript meets PLOS ONE's style requirements, including those for file naming. The PLOS ONE style templates can be found at

https://journals.plos.org/plosone/s/file?id=wjVg/PLOSOne_formatting_sample_main_body.pdf and

https://journals.plos.org/plosone/s/file?id=ba62/PLOSOne_formatting_sample_title_authors_affiliations.pdf

2. Please include your tables as part of your main manuscript and remove the individual files. Please note that supplementary tables (should remain/ be uploaded) as separate "supporting information" files.

3. We note that the original protocol that you have uploaded as a Supporting Information file contains an institutional logo. As this logo is likely copyrighted, we ask that you please remove it from this file and upload an updated version upon resubmission.

4. If the reviewer comments include a recommendation to cite specific previously published works, please review and evaluate these publications to determine whether they are relevant and should be cited. There is no requirement to cite these works unless the editor has indicated otherwise.

5. Please review your reference list to ensure that it is complete and correct. If you have cited papers that have been retracted, please include the rationale for doing so in the manuscript text, or remove these references and replace them with relevant current references. Any changes to the reference list should be mentioned in the rebuttal letter that accompanies your revised manuscript. If you need to cite a retracted article, indicate the article’s retracted status in the References list and also include a citation and full reference for the retraction notice.

[Note: HTML markup is below. Please do not edit.]

Reviewers' comments:

Reviewer's Responses to Questions

**Comments to the Author**

1. Does the manuscript provide a valid rationale for the proposed study, with clearly identified and justified research questions?

Reviewer #1: Yes

Reviewer #2: Yes

**********

2. Is the protocol technically sound and planned in a manner that will lead to a meaningful outcome and allow testing the stated hypotheses?

Reviewer #1: Yes

Reviewer #2: Yes

**********

3. Is the methodology feasible and described in sufficient detail to allow the work to be replicable?

Reviewer #1: Yes

Reviewer #2: No

**********

4. Have the authors described where all data underlying the findings will be made available when the study is complete?

The PLOS Data policy

Reviewer #1: Yes

Reviewer #2: No

**********

5. Is the manuscript presented in an intelligible fashion and written in standard English?

Reviewer #1: Yes

Reviewer #2: Yes

**********

Please use the space provided to explain your answers to the questions above and, if applicable, provide comments about issues authors must address before this protocol can be accepted for publication. You may also include additional comments for the author, including concerns about research or publication ethics.

You may also provide optional suggestions and comments to authors that they might find helpful in planning their study.

Reviewer #1: This is a very well written paper describing the protocol of a three arm parallel group, observer blinded, superiority randomized controlled trial testing music based intervention for anxiety among mothers of preterm neonates. I have a few suggestions that may improve the future analysis of the study.

The sample size calculation is extremely thorough and well documented. However, the current calculation appears to support a pre post two group comparison of main effects under an ANCOVA framework as implemented in the pwr package. In particular, the function pwr.f2.test() assumes a standard linear model and does not account for within subject correlation that arises in repeated measures designs. For longitudinal studies, power estimates based on this approach can be optimistic and may not accurately reflect the required sample size.

This issue would not arise if the design were strictly a pre-post comparison with a corresponding analysis strategy. However, in lines 495 to 500 there are indications that a linear mixed effects model will be used, including testing for a time by group interaction (also referenced in line 529). The power analysis presented earlier does not correspond to this type of model. For longitudinal designs, one could instead use the R package “longpower” with appropriate parameter specification to estimate power. Typically, the required sample size for detecting a time by group interaction in a three arm trial would be larger.

I suggest that the authors clearly specify in the “Secondary endpoint analysis” section which model corresponds to the powered primary hypothesis and which analyses are not explicitly powered and should therefore be considered secondary or exploratory. This clarification would help readers understand the distinction between the primary analysis and the additional longitudinal modeling described later in the manuscript.

Reviewer #2: Dear Authors,

Thank you for giving me the opportunity to review the article. It is recommended that you clarify whether the article tests a model or discusses research results, and discuss them by integrating them with the findings.

**********

what does this mean?). If published, this will include your full peer review and any attached files.

If you choose “no”, your identity will remain anonymous but your review may still be made public.

**Do you want your identity to be public for this peer review?**  For information about this choice, including consent withdrawal, please see our Privacy Policy

Reviewer #1: No

Reviewer #2: No

**********

[NOTE: If reviewer comments were submitted as an attachment file, they will be attached to this email and accessible via the submission site. Please log into your account, locate the manuscript record, and check for the action link "View Attachments". If this link does not appear, there are no attachment files.]

To ensure your figures meet our technical requirements, please review our figure guidelines: https://journals.plos.org/plosone/s/figures

You may also use PLOS’s free figure tool, NAAS, to help you prepare publication quality figures: https://journals.plos.org/plosone/s/figures#loc-tools-for-figure-preparation.

NAAS will assess whether your figures meet our technical requirements by comparing each figure against our figure specifications.

# Author comment: Author response to Decision Letter 1

**DOI:** 10.1371/journal.pone.0360324.r002

3 Aug 2026

Manuscript: PONE-D-25-68653

Effects of music medicine on anxiety among primigravida mothers of preterm neonates: The CalM MOM randomised controlled trial protocol

We sincerely thank the Editor and both the Reviewers for their thoughtful comments and the opportunity to revise our manuscript. We greatly appreciate the time and effort invested in reviewing our work. We have carefully addressed all comments, and the revisions have substantially improved the quality and clarity of the manuscript! The review process has also been a valuable learning experience for us, and we are grateful for your constructive feedback!

Academic Editor Comments (Prof. Sascha Köpke)

AE1. Abstract: The abstract is definitely the weakest part of this protocol, please out more effort in it to optimize the paper. The abstract should be shortened and restructured. Please be more concise and avoid repetitions e.g. on the trial being the first in India. Therefore, most of the text at the end of the abstract starting with “This is the first RCT from India…” can be deleted, including the mention of the UNSDGs. On the other hand, important information e.g. concerning randomization and allocation concealment is missing. Please check e.g. the CONSORT guideline for abstracts.

Response:

We thank the Academic Editor for this comment. The abstract has been substantially restructured. And we have removed the repetitive sentences beginning “This is the first RCT from India…” and the reference to UN SDGs.

The following mandatory CONSORT 2025 abstract elements have been added or clarified:

✅Randomisation:✅Allocation concealment✅Blinding✅Revised Sample size

Background: Preterm birth and Neonatal Intensive Care Unit (NICU) admission are significant stressors that heighten maternal anxiety. Although RCTs have reported that music-based interventions used alongside usual care (UC) are effective and low-cost in reducing maternal anxiety, evidence from India remains limited.

Objective: Primary objective is to assess the impact of listening to recorded classical Indian Instrumental Saraswati Veena music (Ragas Kalyani and Hindolam) (30-minutes/day over 7-days) alongside UC on maternal anxiety (STAI-state anxiety) of primigravida women with preterm neonates in the NICU. Secondary objectives measure changes in anxiety (Perinatal Anxiety Screening Scale (PASS)), maternal-infant bonding, breast milk volume, and neonatal body weight.

Method: This three-arm parallel-group, superiority RCT among primigravida mothers with anxiety (screened using PASS) of preterm neonates (30-36 weeks of gestation, admitted to NICU) delivered vaginally at Government Lady Goschen Hospital, Mangalore. The intervention arm will receive Music Medicine (MM), the comparator arm Nature Sounds (NS), and the control arm UC. Participant blinding is not feasible (auditory intervention), and they serve as their own outcome-assessors (self-reported questionnaires); investigators and the independent statistician will remain blinded. Personnel interacting with participants are blinded to study objectives; enrolment/allocation staff blinded to the randomization sequence, and assessment staff blinded to group identity.

Randomization will employ a STATA-generated, permuted stratified block design stratified by baseline anxiety severity and gestational age (Allocation concealment: Sequentially Numbered, Opaque, Sealed Envelopes; Allocation Ratio: 1:1:1). Sample size (66/arm; total N=198) was determined through a simulation-based power analysis (5,000 iterations, base-R), assuming an MCID of 4 (MM) and 2 (NS) on the STAI, residual SD of ~8, powered at 80% (α=0.05). Blinded sample-size re-estimation will be conducted at 50% enrollment completion. Primary powered analysis will employ ANCOVA (baseline-adjusted), and Secondary (exploratory) analyses will utilize a linear mixed-effects model for longitudinal continuous data with time × group interaction.

AE2a. Methods (a): How will you avoid contamination between groups?

Response: Thanks so much for raising this important point! We have added ‘Strategies to Minimise Contamination Between Study Arms’ (Line 445-466) under Intervention details’.

Strategies to minimise contamination between study arms

As the study will be conducted in a resource-limited setting, dedicated facilities to accommodate participants from different study arms in separate rooms will not be available, and mothers will continue to reside in the same postnatal wards. However, to minimise contamination between the three arms, the following strategies will be implemented:

Private study setting: All 30-minute intervention sessions (Music Medicine and Nature Sounds) will be conducted in a designated quiet private room separate from the shared wards. Participants in the usual care arm will receive no audio intervention.

Individual staff supervision: An independent hospital staff member, masked to the study objectives, will escort each participant to the intervention room, activate only the arm-specific audio file on the laptop, and then leave the room. The RA will conduct pre- and post-session assessments but will not be present during intervention delivery.

Sequential scheduling: As participants from different study arms reside in the same postnatal wards, intervention sessions will be individually scheduled and conducted sequentially to minimise contact between participants undergoing different interventions. Real-time coordination will be maintained through recruitment and intervention logs.

Participant instructions: During the informed consent process, participants will be explicitly instructed not to discuss the content or details of their sessions with other mothers in the ward.

Fidelity documentation: Monitoring charts (Supplementary File 7) will record the exact session time, decibel level, and speaker-to-participant distance for every intervention session to ensure protocol adherence.

AE2b. Methods (b): L. 151: “Stratified random sampling” is mentioned (l. 151), but strata are not reported.

Response: Thank you for pointing this out. In Line 158 we have clarified this as ‘stratified on baseline anxiety severity and gestational age (dichotomised).’

AE2c. Methods (c): L. 317: “professors cum professional musicians”, do you mean “with”?

Response: Thank you for pointing this out! Yes! They are both professors and professional musicians. I have replaced ‘cum’ with ‘and’.

AE2d. Methods (d) L. 465: “Mothers will be screened from the nurse’s record book.” What is meant be screening here? Is this really about “Data collection“?

Response: We thank the editor for pointing out this ambiguity. We agree the sentence was redundant with the eligibility screening process already described in the same paragraph and have removed it to avoid confusion between recruitment/screening and data collection.

AE3a Discussion: (a) What is the probability of the mothers staying in hospital for all 7 days? Is this common? Could this be a problem?

Response: As per the Facility-Based Newborn Care guidelines of the Ministry of Health and Family Welfare, Government of India, preterm infants are discharged only after achieving clinical stability, establishing adequate feeding, and demonstrating sustained weight gain. In our setting, it is common for preterm neonates to remain hospitalized for at least two weeks, until they reach an appropriate discharge weight (typically around 1.8 kg), are off all antibiotics, are taking full oral feeds, and the mother is confident in caring for her infant. Therefore, the probability of mothers remaining available for the full 7-day intervention period is expected to be high. We do not anticipate substantial attrition due to early neonatal discharge; however, any unexpected maternal or neonatal discharge will be documented and accounted for in the analysis. : https://www.iapsm.org/pdf/Guidelines/RMNCH%2BA/Guidelines%20for%20Facility%20Based%20Newborn%20Care%202011.pdf

AE3b. Please discuss why there is no patient and/or public involvement in this study.

Response: We thank the reviewer for encouraging us to consider this, which will improve the trial. Patient and public involvement is incorporated via a Lay/Patient Representative (a mother with lived experience of preterm birth) serving as an independent member of the Trial Steering Committee, providing patient perspective on trial oversight and major decisions. This has been clarified in Supplemental File 8.

Journal Requirements

JR1. Please ensure that your manuscript meets PLOS ONE's style requirements, including those for file naming. The PLOS ONE style templates can be found at https://journals.plos.org/plosone/s/file?id=wjVg/PLOSOne_formatting_sample_main_body.pdf and https://journals.plos.org/plosone/s/file?id=ba62/PLOSOne_formatting_sample_title_authors_affiliations.pdf

Response: Thank you! We have followed this style!

JR2. Please include your tables as part of your main manuscript and remove the individual files. Please note that supplementary tables (should remain/ be uploaded) as separate "supporting information" files.

Response: Thank you! We have included the tables as part of the main manuscript and have removed the individual files.

JR3. We note that the original protocol that you have uploaded as a Supporting Information file contains an institutional logo. As this logo is likely copyrighted, we ask that you please remove it from this file and upload an updated version upon resubmission.

Response: We have removed the MAHE Kasturba Medical College logo from the Supplemental File 9

JR4. If the reviewer comments include a recommendation to cite specific previously published works, please review and evaluate these publications to determine whether they are relevant and should be cited. There is no requirement to cite these works unless the editor has indicated otherwise.

Response: Not applicable.

JR5. Please review your reference list to ensure that it is complete and correct. If you have cited papers that have been retracted, please include the rationale for doing so in the manuscript text, or remove these references and replace them with relevant current references. Any changes to the reference list should be mentioned in the rebuttal letter that accompanies your revised manuscript. If you need to cite a retracted article, indicate the article’s retracted status in the References list and also include a citation and full reference for the retraction notice.

Response: Not applicable

Reviewer 1 Comments

This is a very well written paper describing the protocol of a three arm parallel group, observer blinded, superiority randomized controlled

trial testing music based intervention for anxiety among mothers of preterm neonates. I have a few suggestions that may improve the future analysis of

the study.

Response: Thanks very much for your kind comments! We really appreciate it!

R1-1. The sample size calculation is extremely thorough and well documented. However, the current calculation appears to support a pre post two group comparison of main effects under an ANCOVA framework as implemented in the pwr package. In particular, the function pwr.f2.test() assumes a standard linear model and does not account for within subject correlation that arises in repeated measures designs. For longitudinal studies, power estimates based on this approach can be optimistic and may not accurately reflect the required sample size. This issue would not arise if the design were strictly a pre-post comparison with a corresponding analysis strategy. However, in lines 495 to 500 there are indications that a linear mixed effects model will be used, including testing for a time by group interaction (also referenced in line 529). The power analysis presented earlier does not correspond to this type of model. For longitudinal designs, one could instead use the R package “longpower” with appropriate parameter specification to estimate power. Typically, the required sample size for detecting a time by group interaction in a three arm trial would be larger.

Response: We thank the reviewer for this suggestion! We fully accept the point.

Clarification: (Line 227)

Simulation-based sample size estimation

Primary powered analysis: ANCOVA on STAI change from baseline to Day 7 (adjusting for baseline).

Secondary/exploratory: A linear mixed-effects model for analysing longitudinal continuous data — with time × group interaction (no formal power estimation for secondary exploratory endpoints)

Sample Size Justification – Simulation-Based Power Analysis (Evidence in Governance and Politics (EGAP) methodology)

We conducted a transparent simulation-based power analysis (5,000 iterations) for the primary ANCOVA contrast (Music vs Nature sounds vs Control, Minimal Clinically Important Difference (MCID) = 4 points for Music Medicine, MCID=2 points for Nature Sounds, residual standard deviation (SD) ≈ 8, baseline adjustment).

Complete R Code Used for Revised Power Calculation (Base R – EGAP style)

The following self-contained base-R script was used to generate the power estimates above:

set.seed(123)

possible_n_per_arm <- seq(20, 100, by = 2)

powers <- rep(NA, length(possible_n_per_arm))

alpha <- 0.05; sims <- 5000

for (j in 1:length(possible_n_per_arm)) {

n_per_arm <- possible_n_per_arm[j]; N <- n_per_arm * 3

significant <- rep(NA, sims)

for (i in 1:sims) {

baseline <- rnorm(N, 45, 10)

Y0 <- baseline + rnorm(N, 0, 8)          # Control

Y_music <- Y0 - 4; Y_nature <- Y0 - 2   # Effects (no direct reference: Nature Sounds comparator would have roughly half the effect of the Music Medicine intervention, will be revised during SSR)

Z <- sample(c("Control","Music","Nature"), N, TRUE, c(1/3,1/3,1/3))

Y <- ifelse(Z=="Music", Y_music, ifelse(Z=="Nature", Y_nature, Y0))

fit <- lm(Y ~ Z + baseline)

p <- summary(fit)$coefficients["ZMusic", "Pr(>|t|)"]

significant[i] <- (p <= alpha)

}

powers[j] <- mean(significant)

}

data.frame(n_per_arm = possible_n_per_arm, power = round(powers, 3))

And Line 266 we have added a Blinded Sample Size Re-estimation (BSSR) Plan & Recruitment Forecast

R1-2. I suggest that the authors clearly specify in the “Secondary endpoint analysis” section which model corresponds to the powered primary hypothesis and which analyses are not explicitly powered and should therefore be considered secondary or exploratory. This clarification would help readers understand the distinction between the primary analysis and the additional longitudinal modeling described later in the manuscript.

Response: Thanks very much for pointint this out! We really appreciate it! We have now revised the Statistical Analysis section with two clear subsections:

Line: 227

Simulation-based sample size estimation

Primary powered analysis: ANCOVA on STAI change from baseline to Day 7 (adjusting for baseline).

Secondary/exploratory: A linear mixed-effects model for analysing longitudinal continuous data — with time × group interaction (no formal power estimation for secondary exploratory endpoints)

Line 549

Primary Endpoint Analysis

The primary outcome is the change in STAI state anxiety score from baseline (Day 1 pre-intervention) to Day 7 post-intervention.

The primary analysis will be an ANCOVA comparing the mean change in STAI scores between the three groups, adjusting for baseline STAI score. This analysis corresponds to the pre-specified powered primary hypothesis.

Assumption checking and alternative analysis: Prior to analysis, normality of residuals and homogeneity of variances will be assessed. If these assumptions are violated, the Van Elteren test (stratified Wilcoxon rank-sum test) will be used as the primary non-parametric analysis, stratified by baseline anxiety level and gestational age (dichotomised at the median or clinically relevant cut-off).

If the overall test is statistically significant, post-hoc pairwise comparisons will be performed using Bonferroni correction.

Secondary Endpoint Analyses

All secondary endpoint analyses are considered exploratory. No adjustment for multiplicity will be applied.

Perinatal Anxiety Screening Scale: A mixed-effects model for repeated measures (MMRM) or repeated measures ANCOVA will be used to compare trajectories over time (Days 1, 3, 5, and 7), adjusting for baseline score where appropriate. Group × time interaction will be explored.

Postpartum Bonding Questionnaire: ANCOVA or MMRM will be used to compare total and subscale scores, with adjustment for baseline score if relevant.

Breast Milk Volume (Day 2 and Day 7): ANCOVA on Day 7 values adjusting for Day 2 measurement, or MMRM if appropriate.

Neonatal Body Weight (Day 1 and Day 7): ANCOVA on Day 7 weight, adjusting for baseline weight and relevant covariates (e.g., gestational age at birth).

Vital Parameters (Blood Pressure, Pulse Rate, Respiratory Rate): Within-group pre-post changes will be assessed using paired t-tests. Between-group comparisons at individual time points will use ANOVA or ANCOVA. Time-by-group interaction effects will be explored using MMRM. These analyses are exploratory.

Reviewer 2

R2-1. Dear Authors, Thank you for giving me the opportunity to review the article. It is recommended that you clarify whether the article tests a model or discusses research results, and discuss them by integrating them with the findings.

Response: Thank you! This is a protocol of a randomised controlled trial. To clarify this we have written ‘Protocol’ in the title and we have used future tense throughout the manuscript.

Attachment Submitted filename: Response_to_Reviewers_PONE-D-25-68653.docx

# Editor report: Decision Letter 1

**DOI:** 10.1371/journal.pone.0360324.r003

**Authors:** Sascha Köpke

15 Sep 2026

<p>Effects of music medicine on anxiety among primigravida mothers of preterm neonates: The CalM MOM randomised controlled trial protocol

PONE-D-25-68653R1

Dear Dr. Kamath,

We’re pleased to inform you that your manuscript has been judged scientifically suitable for publication and will be formally accepted for publication once it meets all outstanding technical requirements.

Within one week, you’ll receive an e-mail detailing the required amendments. When these have been addressed, you’ll receive a formal acceptance letter and your manuscript will be scheduled for publication.

An invoice will be generated when your article is formally accepted. Please note, if your institution has a publishing partnership with PLOS and your article meets the relevant criteria, all or part of your publication costs will be covered. Please make sure your user information is up-to-date by logging into Editorial Manager at Editorial Manager® and clicking the ‘Update My Information' link at the top of the page. For questions related to billing, please contact billing support.

If your institution or institutions have a press office, please notify them about your upcoming paper to help maximize its impact. If they’ll be preparing press materials, please inform our press team as soon as possible -- no later than 48 hours after receiving the formal acceptance. Your manuscript will remain under strict press embargo until 2 pm Eastern Time on the date of publication. For more information, please contact onepress@plos.org.

Kind regards,

Sascha Köpke

Academic Editor

PLOS One

Additional Editor Comments (optional):

Reviewers' comments:

# Editor report: Acceptance letter

**DOI:** 10.1371/journal.pone.0360324.r004

**Authors:** Sascha Köpke

PONE-D-25-68653R1

PLOS One

Dear Dr. Kamath,

I'm pleased to inform you that your manuscript has been deemed suitable for publication in PLOS One. Congratulations! Your manuscript is now being handed over to our production team.

At this stage, our production department will prepare your paper for publication. This includes ensuring the following:

* All references, tables, and figures are properly cited

* All relevant supporting information is included in the manuscript submission,

* There are no issues that prevent the paper from being properly typeset

You will receive further instructions from the production team, including instructions on how to review your proof when it is ready. Please keep in mind that we are working through a large volume of accepted articles, so please give us a few days to review your paper and let you know the next and final steps.

Lastly, if your institution or institutions have a press office, please let them know about your upcoming paper now to help maximize its impact. If they'll be preparing press materials, please inform our press team within the next 48 hours. Your manuscript will remain under strict press embargo until 2 pm Eastern Time on the date of publication. For more information, please contact onepress@plos.org.

You will receive an invoice from PLOS for your publication fee after your manuscript has reached the completed accept phase. If you receive an email requesting payment before acceptance or for any other service, this may be a phishing scheme. Learn how to identify phishing emails and protect your accounts at https://explore.plos.org/phishing.

If we can help with anything else, please email us at customercare@plos.org.

Thank you for submitting your work to PLOS One and supporting open access.

Kind regards,

PLOS One Editorial Office Staff

on behalf of

Professor Sascha Köpke

Academic Editor

PLOS One
